# Machine Learning in Rugby Union: Predicting and Identifying Key Performance Indicators for Professional Rugby Union Players in Match Play Based Workload

**DOI:** 10.1002/ejsc.70042

**Published:** 2025-08-22

**Authors:** Xiangyu Ren, Simon Boisbluche, Kilian Philippe, Mathieu Demy, Sami Äyrämö, Ilkka Rautiainen, Shuzhe Ding, Jacques Prioux

**Affiliations:** ^1^ Sino‐French Joint Research Center of Sport Science Key Laboratory of Adolescent Health Assessment and Exercise Intervention of Ministry of Education College of Physical Education and Health East China Normal University Shanghai China; ^2^ Movement, Sport, Health Laboratory University of Rennes 2 Bruz France; ^3^ Department of Sports Sciences and Physical Education École Normale Supérieure de Rennes Bruz France; ^4^ Rugby Club Vannes French Rugby Federation Vannes France; ^5^ Laboratory of Movement, Balance, Performance and Health (MEPS, EA‐4445) University of Pau and Pays de l'Adour Tarbes France; ^6^ Faculty of Information Technology University of Jyväskylä Jyväskylä Finland; ^7^ Wellbeing Services County of Central Finland Jyväskylä Finland

**Keywords:** performance optimization, rugby training, team sports, time‐motion analysis, video analysis, workload monitoring

## Abstract

Rugby union is an intermittent high‐intensity contact sport requiring the analysis of various training and match metrics. Time‐motion analysis and video analysis have enhanced the understanding of the interplay between these two factors. However, limited studies have investigated the effect of workload on key performance indicators (KPIs) during matches. In this study, data collected from the global positioning system (GPS) were used to calculate cumulative workload values over 7, 14, and 21 days prior to each game. After dimensionality reduction through principal component analysis (PCA), these workload values were employed as features, with game KPIs as target variables. Modeling was conducted using linear regression (LR), support vector regression (SVR), random forest regression (RFR), and light gradient boosting machine (LightGBM) for regression tasks. The superiority of the model was assessed by coefficient of determination ($R^{2}$), root mean square error (RMSE), and correlation coefficient (R). The findings revealed that although individual GPS metrics exhibited weak correlations with KPIs, machine learning (ML) models particularly RFR, successfully captured complex interactions and nonlinear relationships. These models achieved significantly improved predictive performance, with $R^{2}$ values ranging from 0.40 to 0.72 for certain KPIs. Using SHapley Additive exPlanations (SHAP) analysis and partial dependence plots, this study enhanced the interpretability of ML models by identifying the influence of GPS features on KPIs and exploring their underlying mechanisms. These findings offer actionable insights for workload management, emphasizing critical factors that affect player performance.

## Highlights

- Machine learning improved prediction accuracy compared to single‐feature correlation, with random forest performing best overall.
- Key workload features influenced different KPIs. For forwards, training load and heart rate exertion were most important for tackles and carries. For backs, sprinting intensity and deceleration metrics strongly impacted passes, kicks, and receipts.

## 1 Introduction

In rugby union, both during training sessions and competitive match‐play, players engage in a diverse range of physical activities. These include high‐intensity actions, such as collisions, accelerations, and directional changes, alongside low‐intensity movements such as jogging and walking (Roberts et al. 2008). To effectively manage these varied demands, analyzing workload is essential. Workload refers to the comprehensive measurement of the physical and psychological demands placed on an athlete, which is crucial for understanding the dose‐response relationship between stress and internal responses (Bourdon et al. 2017; Impellizzeri et al. 2023). It is typically divided into two components: external workload and internal workload. External workload quantifies the work completed by an athlete independently of their internal characteristics (Wallace et al. 2009). In contrast, internal workload consists of the athlete's psychophysiological responses, including heart rate (HR) and perceived exertion, which occur while executing the exercises prescribed by the coach (Impellizzeri et al. 2019).

At high levels of competitive sport, coaches and sports scientists are constantly seeking new ways to assess a player's performance to gain an advantage over their opponents. To assess the athletic demands and quantify the performance of professional players and matches in rugby union, two primary methods are currently employed (Cunningham et al. 2018). First, global positioning system (GPS) devices are used to perform time‐motion analyses, enabling coaches to quantify external workload. These quantified data include key metrics such as distance covered, speed, acceleration, and deceleration of the players (Deutsch et al. 2007). Second, video footage of matches is collected and manually coded by trained video analysts based on visual observation. These coded events are used to generate key performance indicators (KPIs), such as tackles, passes, turnovers, possessions, and kicks (Watson et al. 2017). Together, these two methods aim to encode the relevant technical and tactical behaviors and running activities of players in actual playing environments, such as training sessions or matches, providing crucial data to optimize player performance (Jones et al. 2004; Ungureanu et al. 2021; Ungureanu et al. 2019).

KPIs are defined as a selection or combination of action variables that aim to represent some or all aspects of performance, facilitating objective quantification of performance (Parmar et al. 2017). In recent years, researchers have analyzed the relationship between multiple KPIs and the outcomes of elite‐level rugby union matches. They found that winning teams had fewer passes and turnovers when in possession compared to their opponents (Vaz et al. 2010). Indicators contributing to team success include lineouts won on opposition throws and tries scored, which are significantly higher in winning teams compared to losing teams (Kohavi 1995). From an attacking perspective, teams that carried the ball further per possession and achieved a greater number of clean breaks were more likely to win (Watson et al. 2017). In the southern hemisphere professional rugby union, winning teams tend to reduce the number of rucks and passes, kick for more possession, and make more tackles (Vaz et al. 2010). In elite rugby union, winning teams demonstrate a higher number of successful tackles (Ortega et al. 2009) and a reduced frequency of tackle situations (van Rooyen et al. 2014).

In addition, data mining can uncover valuable insights from massive amounts of data, and regression machine learning (ML) models, in particular, have been widely used for prediction across various sports (Bartlett et al. 2017; Bongiovanni et al. 2021; Cornforth et al. 2015; Parmar et al. 2017). These prediction models not only predict the match outcomes (Tümer et al. 2022) but also delve deeper into dimensions such as players' physical condition (Zhou et al. 2017), tactical execution (Cintia et al. 2016), and psychological state (Campo et al. 2019; Jaspers et al. 2018). Coaches can use these techniques to assess training effectiveness, identify strengths and weaknesses during matches, and develop more evidence‐based training plans and tactical arrangements to improve overall performance at both individual and team levels (Bartlett et al. 2017).

As inferred from the above, KPIs are vital metrics for assessing team performance. For coaches and related practitioners striving to enhance match performance, comprehending the significance of workload in achieving success is of paramount importance (Drew et al. 2017). ML techniques are instrumental in such analyses as they enable the development of predictive models, efficiently manage numerous variables, identify nonlinear relationships between workload and KPIs, and determine the most relevant features (Bunker and Thabtah 2019; Cai et al. 2018; Mandorino et al. 2022). Additionally, principal component analysis (PCA) has been applied for dimensionality reduction, helping manage high‐dimensional workload data and improve the efficiency of the predictive models (Carey et al. 2018; Wold et al. 1987). To the author's knowledge, although there have been some studies that have quantified the correlation between workload and performance in team sports (Fox et al. 2018), no previous research has explored the use of ML models to investigate the effects of workload on KPIs in professional rugby union players.

Therefore, the primary objective of this study was to employ ML models to compare and analyze the performance of different algorithms, thereby selecting the optimal model for predicting and interpreting the relationship between dimensionally reduced workload metrics and KPIs. Subsequently, to interpret the impact of principal component (PC) on KPIs, SHapley Additive exPlanations (SHAP) analysis was used, enabling a clearer understanding of each metric's contribution to the predictive models. Finally, we hypothesized that the specific workload metrics derived from PCA identified through the SHAP analysis would significantly contribute to KPIs. It is expected that SHAP values will reveal the most influential metrics in predicting KPI outcomes, thus providing insights into how workload affects performance (de Leeuw et al. 2022).

## 2 Methods

### 2.1 Experimental Approach to the Problem

**FIGURE 1:**
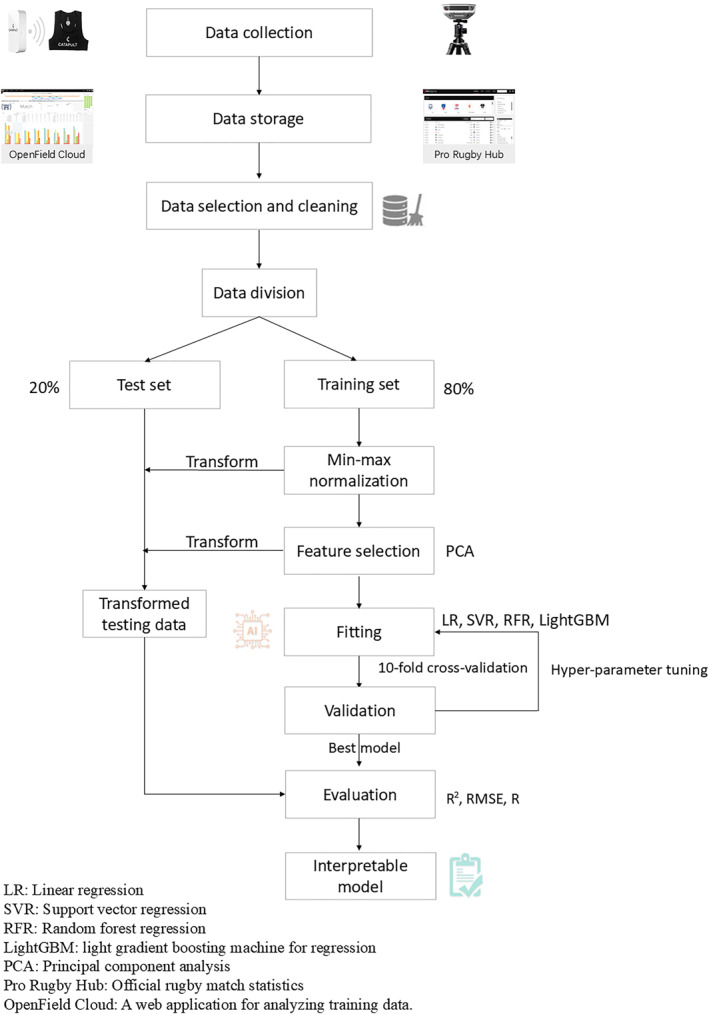
Flowchart of the player KPI performance prediction model.

Considering the workload during the match itself, along with the cumulative workload from the match week (7 days), the previous week (14 days), and the preceding 2 weeks (21 days), where cumulative workload accounts solely for training sessions, we analyzed all matches from the 2021 to 2022, 2022 to 2023, and 2023 to 2024 seasons to clarify the impact of match day and cumulative workload on KPIs. In screening the players' data, we standardized the KPIs to 10 min intervals to ensure the uniformity and rigor of the measurements. During the filtering of independent variables, metrics with a variance inflation factor (VIF) greater than 10 or a high correlation (above 0.9) were excluded to address multicollinearity (Akinwande et al. 2015). After this selection, 18 GPS metrics were retained from the original 104 metrics. For the selection of dependent variables (KPIs), we kept 8 continuous variables from 152 indicators. The overall procedure of this study is shown in Figure 1.

### 2.2 Participants

Data were collected from a cohort of 63 male professional rugby union players (age: 25.7 ± 5.1 years; height: 190.0 ± 10.0 cm; and weight: 103.4 ± 15.8 kg) from the same team (French second division rugby championship, Pro D2). Players were divided into forwards and backs to account for positional differences. All players were familiarized with all monitoring processes. Prior to signing the informed consent form in accordance with the Declaration of Helsinki, the players were informed of the potential benefits and risks of the study with a clear indication of their willingness to share the data collected as part of their daily training practice. The study protocol was conducted with the support of the medical and technical staff of the professional team. Additionally, the study adhered to the ethical guidelines of the university and the research laboratory associated with this study.

### 2.3 Procedures

#### 2.3.1 Workload Monitoring

Workload was captured using a GPS device (Vector X7 sensor, Catapult Innovations, Australia) with integrated 10 Hz GPS, 100 Hz triaxial accelerometer, gyroscope, and 100 Hz magnetometer. To ensure optimal GPS connectivity, the device was activated 30 min before field training in an open area. Each player wore a specialized vest in which sensors (81 × 43 × 16 mm and weighing 53 g) were embedded, positioned on the upper thoracic spine between the shoulder blades. The effectiveness of this device for monitoring running and acceleration metrics in team sports has been validated in previous studies with high reliability (Clavel et al. 2022; Crang et al. 2022). Player load is a metric calculated based on data from triaxial accelerometers, which quantifies the instantaneous rate of change of a player's acceleration in the *X*, *Y*, and *Z* directions. This value is calculated by dividing the square root of these rates of change by 100 (Bredt et al. 2020). GPS and inertial data were exported using the specialized GPS software (Openfield Console 3.7) and stored in OpenField Cloud for further analysis. Table 1 describes the data collected by GPS after selection.

**TABLE 1:** Set of features and labels.

	Variables	Units	Definition
Feature	Player load (PL)	Arbitrary unit	A modified vector magnitude expressed as the square root of the sum of the squared instantaneous rates of change in acceleration in each of the three orthogonal planes and divided by 10.
	Repeated high‐intensity efforts (RHIE)	Number	Three consecutive high‐intensity efforts (contact, acceleration, or sprint) occurring within 21 s.
	Total distance (TD)	Meter	Assessed from GPS, correspond to the total distance covered by the players during the ball‐in‐play time of training.
	Medium‐speed running (MSR)	Meter	Distance covered between 15 and 18 km·h^−1^.
	High‐speed running (HSR)	Meter	Distance covered between 18 and 21 km·h^−1^.
	Very high‐speed running (VHSR)	Meter	Distance covered between 21 and 25 km·h^−1^.
	Sprint running (SR)	Meter	Distance covered above 25 km·h^−1^.
	Acceleration zone 1 (AZ1)	Number	The number of accelerations between 2 and 2.5 m·s^−2^.
	Acceleration zone 2 (AZ2)	Number	The number of accelerations between 2.5 and 3m·s^−2^.
	Acceleration zone 3 (AZ3)	Number	The number of accelerations above 3 m·s^−2^.
	Deceleration zone 1 (DZ1)	Number	The number of decelerations between 2 and 2.5 m·s^−2^.
	Deceleration zone 2 (DZ2)	Number	The number of decelerations between 2.5 and 3m·s^−2^.
	Deceleration zone 3 (DZ3)	Number	The number of decelerations above 3 m·s^−2^.
	Acceleration distance zone 1 (ADZ1)	Meter	Distance at acceleration of 2–2.5 m·s^−2^.
	Acceleration distance zone 2 (ADZ2)	Meter	Distance at acceleration of 2.5–3 m·s^−2^.
	Acceleration distance zone 3 (ADZ3)	Meter	Distance at acceleration of above 3 m·s^−2^.
	Contact involvement total count	Number	Total number of contact involvements in an activity or period that have a duration within contact involvement duration band settings.
	Heart rate (HR) exertion		A weighted score representing total cardiovascular load during exercise, calculated by multiplying time spent in different HR zones by weighted factors.
Label	Total complete tackle	Number	An event where a player carrying the ball (the ball‐carrier) is physically impeded by another player (the tackler).
	Carries	Number	Counts of times the player being in possession of the ball when being tackled by a defending player and included instances whereby the ball carrier offloaded the ball in the process of being tackled.
	Meters carried	Meter	Total meters carried past the gain line.
	Total kicks	Number	The total number of kicks made by players throughout the match.
	Kick meters	Meter	The distance a player kicks during a match.
	Total passes	Number	Counts of times the player passes the ball.
	Total OOA	Number	The total number of arrivals of all players in the ruck. It includes the arrival frequencies of both offensive and defensive players in the ruck area.
	Total receipts	Number	Total number of times a player successfully receives or catches the ball.

#### 2.3.2 Key Performance Indicators

The actions of each player, both on and around the ball during the match, were encoded into a performance matrix with time and location markers derived from video clips. Opta data from Stats Perform (Pro Rugby Hub) provided KPIs for all selected matches. Opta's team of analysts collected data in real‐time and conducted a series of accuracy checks afterward. Each analyst was required to undergo 3–6 months of structured training before being authorized to handle real‐time game coding. Additionally, Opta regularly monitored the accuracy of each analyst throughout the season. Although no studies have yet been published on the reliability of Opta data in rugby union, its data in football have demonstrated strong reliability, with Cohen's Kappa values ranging between 0.92 and 0.94 (Liu et al. 2013). The definitions of the match KPIs used in this analysis are provided in Table 1. These KPIs were selected as they represent the statistical data included in postgame reports, thereby covering multiple facets of the game.

#### 2.3.3 Data Preprocessing

In the present research, the input features consisted of workload data collected via GPS, whereas the output labels were the KPIs. All input features were derived from 18 GPS external workload metrics. For each metric, cumulative training workload was calculated over three time‐windows: 7, 14, and 21 days prior to each match, resulting in a total of 54 original features. To optimize the performance of the different models, standard preprocessing techniques were used. First, a data cleaning process was conducted. If labels were missing for any reason, the corresponding features were also eliminated to keep the dataset as unbiased as possible (Kang 2013). Ultimately, the dataset used in this study contained a total of 1862 samples. Since the dataset was abnormally distributed, a min‐max normalization method was applied to all features within the training set. This scaling method is designed to map feature values to the range [0, 1], achieved by subtracting each feature value from its minimum value in the training set and dividing this difference by the range (maximum value minus minimum value) of that feature. This normalization method ensured that all features contributed equally to the learning process of the ML models (Mohamad and Usman 2013). Feature selection was implemented as a part of data preprocessing to eliminate irrelevant or redundant subsets of features.

PCA is a widely used approach for exploratory data analysis, feature extraction, and dimensionality reduction. Its goal is to reduce dimensionality and enhance interpretability while preserving critical information. By calculating PCs and using them to capture the core variations in the data, PCA generates a smaller set of low‐redundancy variables, transforming key information from the observed data into a linear combination of orthogonal components, thereby effectively highlighting the primary features of the data (Jolliffe and Cadima 2016). In PCA, the first PC (PC1) that captures maximum variance can be represented as a linear combination:

$$\text{PC}1=\omega_{11}x_{1}+\omega_{11}x_{2}+...+\omega_{1m}x_{m}.$$

where $\omega_{1}$ corresponds to an eigenvector of the covariance matrix:

$$\sum =\frac{1}{\left(N-1\right)}X_{\text{centralized}}^{T}X_{\text{centralized}}.$$

The loadings for the PC1 are computed as follows:

$${\text{Loading}}_{1j}=\sqrt{\lambda_{1}}\cdot \omega_{1j},$$

where $\omega_{1j}$ is the j‐th element of the eigenvector associated with the PC1 and $\lambda_{1}$ is the corresponding eigenvalue (explained variance).

PCA was applied to 54 GPS‐derived workload features in this research. The first 12 PCs for forwards and 13 PCs for backs were retained, as these together explained over 95% of the variance in the data. This indicates that low‐dimensional but high‐fidelity data representation can effectively support feature engineering.

#### 2.3.4 Model Development

Since the output labels of the dataset were determined to be continuous, the task was classified as a regression problem. In this study, we used the Python 3.12 development environment and modeled the data with several different regression algorithms in the scikit‐learn library (version 1.4.2). Before modeling with PCA‐reduced features, we used the correlation R between each individual raw feature and the label as a baseline. For the PCA‐based modeling, we split the data into training and testing sets with 80% of the data used for training and 20% for testing, while employing the test set to evaluate the model's generalization ability without a separate validation set (Kernbach and Staartjes 2022). After selecting the model structure, we applied ten‐fold cross‐validation on the training set to evaluate model performance across different hyperparameter settings using a random search with 200 rounds to fine‐tune the parameters (Kohavi 1995). The linear regression (LR), support vector regression (SVR), random forest regression (RFR), and light gradient boosting machine (LightGBM) algorithms (explicit formulas are provided in the Supporting Information S1) were applied to the dataset, with the specific parameters described in Table 2.

**TABLE 2:** Hyperparameter values for machine learning models considered in this research.

Model	Hyper‐parameter	Meaning	Values
SVR	K	Kernel	[“linear”, “rbf”]
	γ	Kernel coefficient	[“scale”, “auto”]
	C	Regularization parameter	[0.1, 1, 2, 4, 6, 8, 10]
	ε	No penalty associated with points predicted	[0, 0.2, 0.4, 0.6, 0.8, 1]
RF	n_estimators	The number of iterations (number of trees)	[50, 100]
	max_depth	Maximum allowed depth for trees	[1, 3, 5, 7, 9, 11, 13, 15, 17, 19]
	min_samples_split	Number of samples required to split an internal node	[2, 4, 6]
	min_samples_leaf	Minimum number of samples required for leaf nodes	[1, 2, 4]
LightGBM	num_leaves	The number of leaf nodes	[2, 4, 6, 8, 10]
	force_col_wise	Forces column‐wise data storage	True
	learning_rate	Determining the step size in each iteration	[0.02, 0.04, 0.06, 0.08, 0.01]
	n_estimators	The number of iterations (number of trees)	[50, 100]
	min_child_samples	The minimum number of samples required in each leaf node	[2, 4, 6, 8]
	subsample	The proportion of samples used per tree	[0.6, 0.8, 1.0]
	colsample_bytree	The proportion of features used to build each tree	[0.6, 0.8, 1.0]
	reg_alpha	The L1 regularization term	[0, 0.2, 0.4, 0.6, 0.8]
	reg_lambda	The L2 regularization term	[0, 0.2, 0.4, 0.6, 0.8]

Abbreviations: LightGBM, light gradient boosting machine for regression; RF, random forest; SVR, support vector regression.

### 2.4 Statistical Analyses

#### 2.4.1 Performance Evaluations

When evaluating the performance of prediction models, several metrics were used to measure their effectiveness. The coefficient of determination ($R^{2}$) quantifies the proportion of the variance in the actual values that can be explained by the prediction model (Asuero et al. 2006). Root mean squared error (RMSE) is a measure of prediction error, calculated as the square root of the average squared differences between predicted and actual values, and it provides an error metric in the same units as the original data (Willmott and Matsuura 2005). The Pearson correlation coefficient (R) is a measure of the strength of the correlation between the real output series and the predicted output series.

$$R^{2}=1-\frac{\sum_{i=1}^{N}\;{\left({\hat{y}}_{i}-y_{i}\right)}^{2}}{\sum_{i=1}^{N}\;{\left(y_{i}-\bar{y}\right)}^{2}},$$

$$\text{RMSE}=\sqrt{\sum_{i=1}^{N}\;\frac{{\left({\hat{y}}_{i}-y_{i}\right)}^{2}}{N},}$$

$$R=\frac{\sum_{i=1}^{N}\;\left(y_{i}-\bar{y}\right)\left({\hat{y}}_{i}-\bar{\hat{y}}\right)}{\sqrt{\sum_{i=1}^{N}\;{\left(y_{i}-\bar{y}\right)}^{2}}\cdot \sqrt{\sum_{i=1}^{N}\;{\left({\hat{y}}_{i}-\bar{\hat{y}}\right)}^{2}}},$$

where ${\hat{y}}_{i}$ represents the predicted output of the regression model for the i‐th sample, $y_{i}$ is the actual value or expected value for the sample i, $\bar{y}$ denotes the average of the actual values, and $\bar{\hat{y}}$ represents the mean of the predicted values. According to these metrics, a well‐performing regressor is demonstrated by an $R^{2}$ value close to 1, an RMSE close to 0, and an R value near 1 (or −1 for negative correlation).

#### 2.4.2 SHAP Implementation

Feature importance analysis was conducted to identify the features that most significantly influenced the model's predicted outcomes. The SHAP method, proposed by Lundberg and Lee (2017), is an advanced model‐interpretation tool based on game theory (Shapley 1953; Štrumbelj and Kononenko 2014). This approach evaluates the contribution of each feature to the model's output by calculating its marginal effect while considering all possible feature interactions. SHAP not only measures the magnitude of a feature's influence on predictions but also identifies whether its impact is positive or negative.

The SHAP feature contribution formula, based on the Shapley value, is as follows:

$$\phi_{i}=\sum_{S\subseteq F\backslash \left\{i\right\}}\;\frac{\left|S\right|!(|F\left|-\right|S|-1)!}{|F|!}\left[f\left(S\cup \left\{i\right\}\right)\left(x_{S\cup \left\{i\right\}}\right)-f\left(S\right)\left(x_{S}\right)\right],$$

where $\phi_{i}$ represents the SHAP value for feature i, which indicates the marginal contribution of feature i to the model's output. The notation S⊆F\{i} refers to all subsets S that do not include feature i. The expression |S| denotes the number of features in subset S. The term $f\left(S\cup \left\{i\right\}\right)\left(x_{S\cup \left\{i\right\}}\right)-f\left(S\right)\left(x_{S}\right)$ represents the marginal contribution to the model's output when feature i is added to subset S, reflecting the impact of feature i on the model's output.

This study conducted a SHAP analysis to identify the most important features and explore the interactions between PCs and KPIs. SHAP summary plots were generated to visually display the key features. The type of SHAP interpretation applied depended on the model used; for instance, TreeExplainer was employed if a random forest model was found to perform best.

## 3 Results

### 3.1 Comparison of Model Performance

Table 3 summarizes the baseline results for predicting KPI labels using single features for both forwards and backs, with absolute R values ranging from 0.09 to 0.41. Only the results with the highest correlations are presented here; the complete dataset is available in Table S1.

**TABLE 3:** Baseline performance of single‐feature and KPI label correlation coefficients (*R*) for forwards and backs (highest *R* values and corresponding features are displayed).

KPI	Forward		Back	
	Feature	R	Feature	R
Carries	Load_21_Days_DZ3	0.09	Load_14_Days_AZ1	−0.10
Kick meters	Load_21_Days_ADZ1	−0.43	Load_21_Days_SR	−0.36
Meters carried	Load_7_Days_DZ3	0.16	Load_21_Days_MSR	−0.22
Total complete tackles	Load_7_Days_HR exertion	−0.13	Load_21_Days_SR	−0.16
Total kicks	Load_21_Days_HR exertion	−0.36	Load_21_Days_SR	−0.41
Total OOA	Load_14_Days_SR	−0.20	Load_7_Days_Contact involvement total count	0.11
Total passes	Load_7_Days_RHIE	0.15	Load_21_Days_SR	−0.41
Total receipts	Load_14_Days_DZ3	0.14	Load_21_Days_SR	−0.41

Abbreviations: ADZ1, acceleration distance zone 1; AZ1, acceleration zone 1; DZ3, deceleration zone 3; HR, heart rate; KPI, key performance indicator; MSR, medium‐speed running; RHIE, repeated high‐intensity efforts; SR, sprint running, distance covered above 25 km·h^−1^.

**FIGURE 2:**
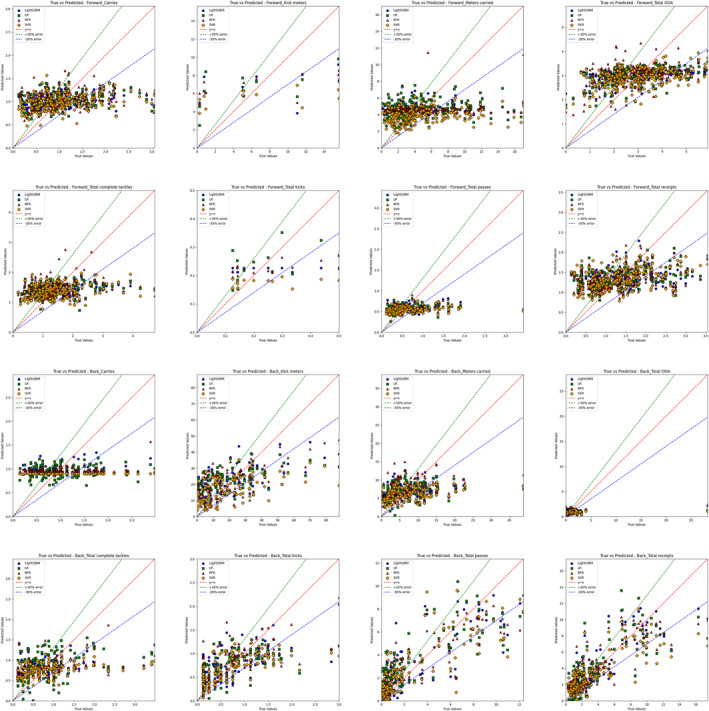
Comparison of predictive values of different machine learning models. An ideal prediction line, marked in red, indicates perfect alignment between predicted and actual values. The green line and the blue line represent the acceptable error range between predicted and true values, with the green line indicating a +30% error range and the blue line indicating a −30% error range.

$R^{2}$, RMSE, and R computed using the actual and predicted values for each algorithm are displayed in Table 4. The R values after ML modeling outperformed the baseline. For example, for backs' total passes, the baseline absolute R value of 0.41 improved to 0.87 with the RFR model. Figure 2 and Figure S1 depict the relationship between actual and predicted values across various regression models, visualized through scatter plots.

**TABLE 4:** Results of multiple machine learning models evaluations.

KPI	Model	Forward			Back		
		$R^{2}$	RMSE	R	$R^{2}$	RMSE	R
Carries	LR	0.04	0.59	0.21	0.00	0.56	0.11
	SVR	0.07	0.59	0.28	−0.03	0.57	0.09
	RFR	0.09	0.58	0.29	0.05	0.55	0.28
	LightGBM	0.05	0.59	0.22	0.04	0.55	0.21
Kick meters	LR	0.26	4.91	0.56	0.21	15.68	0.50
	SVR	0.07	5.50	0.43	0.24	15.36	0.52
	RFR	0.16	5.23	0.52	0.42	13.46	0.68
	LightGBM	0.22	5.06	0.52	0.31	14.64	0.59
Meters carried	LR	0.04	3.42	0.21	0.12	5.28	0.35
	SVR	−0.01	3.51	0.20	0.10	5.34	0.35
	RFR	0.05	3.40	0.23	0.10	5.32	0.35
	LightGBM	0.02	3.45	0.14	0.03	5.54	0.19
Total complete tackles	LR	0.07	0.70	0.26	0.12	0.64	0.37
	SVR	0.06	0.71	0.25	0.12	0.64	0.41
	RFR	0.02	0.72	0.21	0.13	0.64	0.37
	LightGBM	0.04	0.71	0.21	0.05	0.67	0.23
Total kicks	LR	0.26	0.10	0.51	0.31	0.49	0.56
	SVR	−0.25	0.13	0.26	0.34	0.48	0.61
	RFR	0.11	0.11	0.50	0.40	0.46	0.64
	LightGBM	0.00	0.12	0.26	0.37	0.47	0.61
Total OOA	LR	0.15	1.12	0.41	0.02	2.32	0.16
	SVR	0.17	1.11	0.43	0.01	2.33	0.13
	RFR	0.15	1.12	0.39	0.09	2.22	0.42
	LightGBM	0.11	1.15	0.38	0.07	2.25	0.45
Total passes	LR	0.03	0.43	0.19	0.61	2.01	0.78
	SVR	0.05	0.43	0.28	0.66	1.89	0.81
	RFR	0.06	0.42	0.29	0.72	1.70	0.87
	LightGBM	0.05	0.43	0.24	0.70	1.75	0.84
Total receipts	LR	0.14	0.67	0.37	0.59	2.17	0.77
	SVR	0.13	0.68	0.37	0.59	2.15	0.78
	RFR	0.12	0.68	0.35	0.69	1.87	0.84
	LightGBM	0.09	0.69	0.30	0.69	1.86	0.84

Abbreviations: KPI, key performance indicator; LightGBM, light gradient boosting machine for regression; LR, linear regression; R, Pearson correlation coefficient; *R* ^2^, coefficient of determination; RF, random forest; RMSE, root mean squared error; SVR, support vector regression.

In the performance analysis of eight KPIs, differences were observed in the models' effectiveness for forwards and backs. Overall, the models for backs outperformed those for forwards (except for total OOA). For example, in total passes and total receipts, the RFR model performed better in backs than in forwards. Specifically, total passes reaching an $R^{2}$ of 0.72 compared to 0.06 for forwards; total receipts for backs achieved an $R^{2}$ of 0.69, tied for best with LightGBM and higher than the 0.12 for the forward model. For kick meters, total kicks, and total receipts, LR performed well in forward models. Additionally, SVR achieved an $R^{2}$ of 0.17 in forward modeling for total OOA, outperforming other models. However, certain KPIs had generally low predictive power in both positions, including carries, meters carried, and total complete tackles.

### 3.2 SHAP Analysis

**FIGURE 3:**
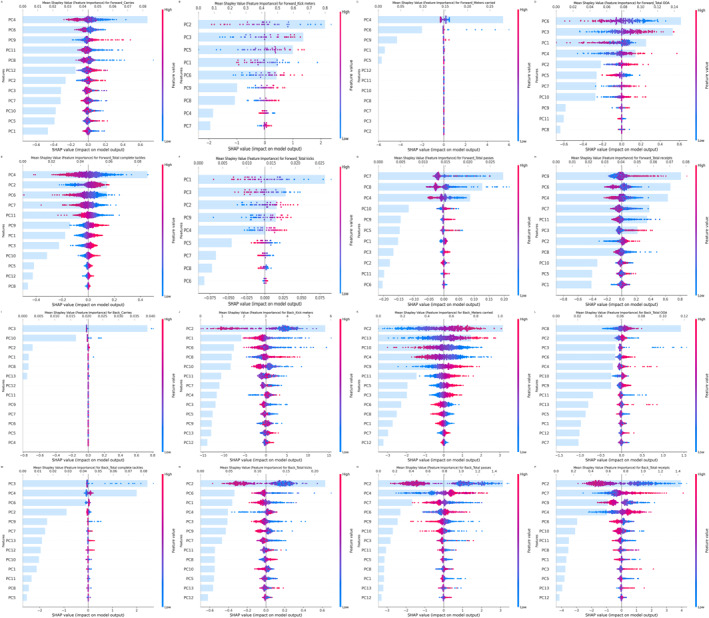
SHAP feature importance summary for KPIs prediction.

The complete data on variance ratio and component loadings for all components can be found in Tables S2 and S3, respectively. Figure 3 presents the summary plot of SHAP feature importance analysis, effectively capturing the relative impact of all features across the dataset. The most important features are those with the largest ranges of SHAP values. Each dot represents the contribution of a feature, where red indicates high SHAP values, signifying a positive contribution of the input parameter to the corresponding output, whereas blue represents low SHAP values, indicating a negative contribution to the output. The accompanying bar chart shows the absolute mean of SHAP values for each feature, providing a distribution of feature importance. This effectively balances the positive and negative impacts observed in the summary plot above, offering a clearer ranking of feature importance.

As shown in Figure 3, for forwards, the top two features for carries were PC4 and PC6, where lower values of these components positively impacted the model output. For total complete tackles, PC4 and PC2 emerged as the most important features, with lower values of PC4 and higher values of PC2 positively influencing the model output. For total OOA, PC6 was identified as the most significant feature, where lower values of this component contributed positively to the predictions. Regarding the component loadings (Table S3), PC4 had substantial contributions from the 7‐day TD and PL as well as the 7‐, 14‐, and 21‐day HR exertion. PC6 was predominantly associated with the 7‐, 14‐, and 21‐day SR metrics, whereas PC2 exhibited stronger contributions from the total contact involvement count across the 7‐, 14‐, and 21‐day periods.

For backs, Figure 3 shows that PC2 and PC6 were the top two features for total kicks, with lower values of these components positively influencing the model output. For total passes and total receipts, PC2 and PC4, PC2 and PC7 were the most important features, where lower values of PC2 and higher values of PC4 contributed positively to the predictions. Component loadings (Table S3) showed that PC2 and PC4 had higher contributions from the 7‐, 14‐, and 21‐day contact involvement total count. However, PC2 was more closely related to running intensity (e.g., SR and VHSR), whereas PC4 focused on acceleration and deceleration metrics. The 7‐, 14‐, and 21‐day VHSR and SR metrics served as primary contributors to PC6. PC7 exhibited stronger loadings from the 7‐, 14‐, and 21‐day HR exertion and deceleration zones (DZ2 and DZ3).

**FIGURE 4:**
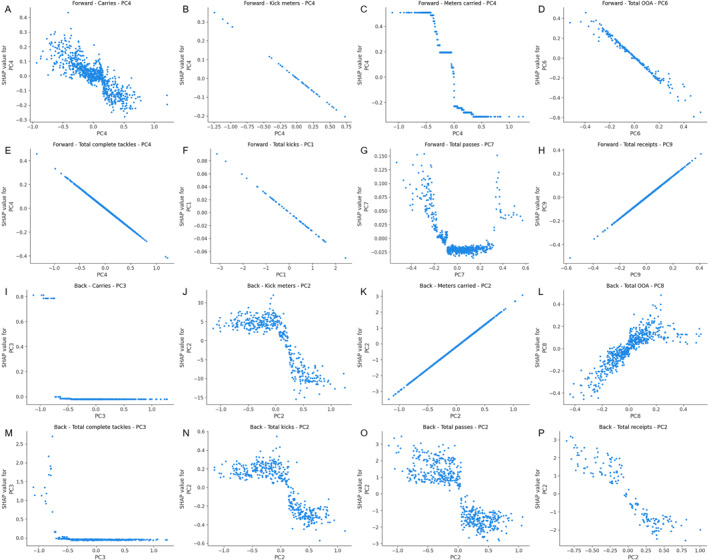
SHAP main effects plots.

Based on the SHAP feature importance analysis derived from the optimal ML model, Figure 4 presents several univariate partial dependence plots. The results showed that for forward carries, lower PC values positively contributed to KPI predictions, increasing the predicted values. Similarly, for back kick meters, total kicks, total passes, and receipts, lower PC values positively influenced KPI predictions, enhancing the predicted outcomes. In contrast, for backs' total OOA, higher PC values positively impacted KPI predictions, increasing the predicted values. Detailed univariate partial dependence plots for all components are included in Figure S2.

## 4 Discussion

To date, the multivariable relationship between workload metrics and KPIs in rugby union has not been studied. Here, our goal was to fill this gap in the literature by using ML regression techniques. First, we adopted an exploratory approach to select the best ML regression model for each KPI for forwards and backs, focusing on the relationship between PC of cumulative workload over different periods (obtained through dimensionality reduction) and KPIs. After identifying the best ML model, we conducted a SHAP feature importance analysis. The interpretability of the ML model enabled us to quantify the importance of each feature and rank them based on their contribution to KPI prediction, providing insights into the independent effects of each feature on the target. Finally, we visualized the univariate dependencies of the most important features on the target variables, providing scientific evidence and practical guidance for optimizing future KPI prediction models and applications.

Considering multiple time scales of cumulative workload is crucial for investigating the relationship between workload and performance in sports. This is because performance and recovery processes are complex systems influenced by various factors such as short‐term or long‐term effects, fatigue accumulation, training cycles, and individual differences (Bowen et al. 2017; Colby et al. 2014; Mohr et al. 2023; Soligard et al. 2016). By examining workload over 7, 14, and 21 days, we expected to gain a more comprehensive understanding of players' overall status and performance levels. In addition, research has shown that analyzing KPIs can be highly beneficial as they are valued by coaches and used to provide feedback on key aspects of the match (Bremner et al. 2013; Colomer et al. 2020). Based on previous research findings, completing more tackles, carries, and kicks while making fewer passes can increase the likelihood of a team winning (Bishop and Barnes 2013; Watson et al. 2017). This finding also explains why these KPIs were prioritized in this research.

In this study, the LR model performed well for forwards in predicting KPIs such as total kicks, kick meters, total complete tackles, and total receipts. However, the $R^{2}$ values remained consistently low, likely due to the model's limited ability to capture the complexity of nonlinear relationships between the independent variables and the dependent variable. For backs, although the RFR model was not the best performer for meters carried and total carries, it demonstrated superior performance for most other KPIs, particularly for total passes and total receipts, where it provided the most accurate predictions. The randomness of the RFR model is derived from two core steps: bootstrap sampling and feature randomization. These mechanisms help reduce overfitting, enhance generalization, and may explain its effectiveness for certain tasks in this study (Breiman 2001). Several studies have also highlighted the effectiveness of the RFR model. For example, Mandorino et al. (2022) found that RFR outperformed other models in predicting soccer players' recovery status using training load data. de Leeuw et al. (2022) reported RFR to be the best predictor of offensive behavior in elite volleyball. These findings support the potential value of ensemble models in sports performance analysis, although in our study, other methods, such as SVR and LightGBM, outperformed RFR in certain KPIs, highlighting that model performance is task‐specific. Although there are few examples of partial dependence plots being used to explain statistics for practical use in sports performance analysis (Bennett et al. 2019; Mosey and Mitchell 2020), their application in the sports field offers coaches and analysts new insights. These plots intuitively illustrate the relationship between features (workload) and targets (KPIs).

Despite the relatively low $R^{2}$ values of many models in this study, their Pearson correlation coefficients were consistently higher than baseline values. This indicates that ML methods exhibit significant potential for uncovering complex relationships between KPIs and workload, offering new possibilities for predictive research in related fields. This study demonstrates that single‐variable models are insufficient for capturing the complexity of the data. By applying PCA for dimensionality reduction, integrated features significantly improved the interpretability and performance of the fitted models. In addition, when performing LR between principal PCs and KPIs to evaluate the explanatory power of individual PCs, correlations lower than those achieved by ML models were observed (Table S4). This indicates that single PCs were insufficient to fully explain the variation in target variables and cannot serve as direct predictors. These findings reflected the presence of more complex nonlinear or high‐dimensional interactions between features and target variables. In such scenarios, ML methods demonstrated significant advantages by effectively capturing these intricate patterns.

In the research by Cousins et al. (2023), players who spent more time on defense were required to make more tackles to stop the opponent's attacks. Conversely, teams that spent more time attacking had more opportunities to carry the ball forward and try to break through the opponent's defense. Therefore, the specific situation and tactical strategy of the match influenced the team's performance. In our research, if more tackles were required, attention should be paid to training metrics, such as TD, PL, total contact involvement count, and HR exertion drills, to ensure optimal team performance and fitness management for forwards. This provides data support for teams to formulate more effective game plans and training strategies. Meanwhile, refining offensive and defensive strategies during matches requires attention not only to selecting appropriate workload ranges based on partial dependence plots but also to players' positions, movement postures, and tackling angles. For example, inside backs and outside backs are more likely to break tackles than tight forwards, and tacklers with an up and forward head position are more successful than those with a moving head position (Hendricks et al. 2014). Additionally, tackling success improves when defenders maintain forward body lean and make contact from frontal or oblique angles rather than from behind (Hendricks et al. 2014). This ensures that players can adapt flexibly to various situations during matches, thereby achieving better match performance (van Rooyen et al. 2014). According to Mosey and Mitchell (2020) and Bennett et al. (2019), the longer a team carries the ball in a match (in meters), the greater its probability of winning. Therefore, if the team aims to increase the number of meters carried during a match, it should focus on mechanical workload metrics, such as deceleration, as well as running workload metrics such as VHSR and SR.

In terms of offense, unsuccessful teams completed more passes (Vaz et al. 2019). The findings of this study revealed that contact involvement total count and ADZ3 were the primary contributors to pass changes among backs during matches. However, this effect largely depended on the tactical strategies employed during the game. To minimize the number of passes, player running ability remained crucial. Teams may tend to create attacking opportunities and space through proactive running during attacks, rather than relying excessively on passing to seek opportunities, thereby improving offensive efficiency and success rate.

In addition, an increase in the number of kicks is associated with an improved likelihood of a positive outcome in the match (Bennett et al. 2019). Particularly in the Rugby World Cup finals, defensive performance often has a greater influence on the game's outcome than offensive performance. When teams adopted a kicking‐based tactic and executed it within an effective defensive structure, they were more likely to win the game rather than relying excessively on possession (Vaz et al. 2019). The present study highlighted the critical role of high‐speed, very‐high‐speed, and sprint running metrics derived from GPS data collected in contributing to the number of kicks, whereas acceleration and deceleration metrics were also shown to be essential factors. This finding aligned with previous studies and emphasized the importance of improving players' strength, explosiveness, aerobic and anaerobic fitness to enhance their speed, change of direction, and other motor abilities. These improvements in fitness are closely linked to a number of KPIs and drive performance coaches and technical coaches to adopt holistic training approaches (Cunningham et al. 2018). Although fitness tests estimate a player's maximal physical potential under controlled conditions, in‐game running metrics reflect the integration of that potential with tactical roles, opposition pressure, and match context. This means that workload, fitness, and tactical technique need to be combined when training rugby union players (Cunningham et al. 2018).

Despite the novelty of this study, we acknowledge some limitations. Kicks are primarily performed by backs, resulting in fewer kicking data points for forwards. Similarly, backs finished fewer carries, total complete tackles, and total OOA. These variations highlight the need for more data to improve the modeling of workload and performance relationships across player positions. Due to limited data, although we categorized player positions into forwards and backs, further divisions are worth exploring. In general, there were significant differences in training and match workload across the forward positions (Yamamoto et al. 2017). Future studies should aim to identify workload combinations optimized for specific player positions in rugby union (Hughes et al. 2012). Additionally, this study primarily focused on the impact of individual or combined KPIs on player performance and match outcomes in rugby union. However, various other contextual factors, such as players' fitness levels, rankings, and weather conditions on the match day, can also significantly affect player performance and match results. Future research should incorporate these contextual factors into the analysis.

## 5 Practical Applications

From this study, the selection of ML models, along with the importance analysis and partial dependence plots provided by SHAP, has revealed key strategies for optimizing sports performance and training. By understanding the impact of different workload metrics on KPIs, coaches and sports analysts can customize training plans to enhance player performance. The integration of these applications into training and game strategies facilitates timely tactical adjustments and ensures optimal utilization of players based on their workload capacity and identified KPIs.

## Author Contributions

All the authors contributed to the conception and design. The first draft of the manuscript was written by X. Ren with support from K. Philippe, S. Äyrämö, I. Rautiainen, and J. Prioux. Material preparation and data collection were handled by S. Boisbluche and M. Demy. Data analysis was conducted by X. Ren, S. Äyrämö, and I. Rautiainen. All authors have reviewed and commented on earlier versions of the manuscript, providing critical feedback that helped complete the research, analysis, and final manuscript.

## Ethics Statement

All players were familiarized with all monitoring processes. Prior to signing the informed consent form in accordance with the Declaration of Helsinki, the players were informed of the potential benefits and risks of the study with a clear indication of their willingness to share the data collected as part of their daily training practice. The study protocol was conducted with the support of the medical and technical staff of the professional team. Additionally, the study adhered to the ethical guidelines of University of Rennes and the research laboratory associated with this study.

## Conflicts of Interest

The authors declare no conflicts of interest.

## Supporting information


Supporting Information S1

**Figure S1**: Comparison of predictive performance of different machine learning models: separate presentation of model results.

**Figure S2**: Univariate partial dependence plots for each principal component.

**Table S1**: Correlation coefficients between single features and KPI labels for forwards and backs.

**Table S2**: The variance ratio of each principal component.

**Table S3**: Component loadings for all components.

**Table S4**: Correlation coefficients between single principal component and KPI labels for forwards and backs.
