# Supplementary material for: Machine Learning in Rugby Union: Predicting and Identifying Key Performance Indicators for Professional Rugby Union Players in Match Play Based Workload
Source: Eur J Sport Sci. 2025 Aug 22;25(9):e70042. doi: 10.1002/ejsc.70042 (PMC12373978; doi:10.1002/ejsc.70042)
Supplement: Supplementary file 1 — Supporting Information S1 [file EJSC-25-e70042-s001.docx]

# Regression Machine learning Algorithms

## Linear Regression

Linear regression (LR) is a statistical technique widely used in quantitative analysis and prediction of numerical variables. It aims to reveal how one or more explanatory variables, also known as independent variables or predictor variables, can linearly affect and predict a response variable, referred to as the dependent variable or target variable (3). When multiple predictor variables are used to predict a single dependent variable, the prediction is based on a linear combination of input variables (multiple regression model), where each input variable is assigned a corresponding weight determined by a specific parameter:

$$\begin{matrix} y\left( x,\beta\right) & =\beta_{0}+\beta_{1}x_{1}+\beta_{2}x_{2}+\cdots+\beta_{m}x_{m} \\ & =\beta_{0}+\sum_{j=1}^{m} \beta_{j}x_{j} \end{matrix}$$

Where $\beta_{j},\forall;j\in0,1,\cdots,m$ represent the parameters of the multiple regression model, $\beta$ is the parameter vector of the model, and $\beta_{0}$ is the intercept term (2). For this study, $y$ represents the predicted KPI, while the $x$ corresponds to the vector of workload metrics. The parameters of a linear regression model were computed using the normal equation, which is based on the least squares method (54).

LR typically minimizes the mean squared error (MSE):

$$\text{MSE}=\frac{1}{N}\sum_{i=1}^{N} \left( y_{i}-y_{i} \right)^{2}$$

## Support Vector Regression

Support vector regression (SVR) originates from support vector machines (SVMs), aiming to find an optimal hyperplane that ensures all training data points are as close to this hyperplane as possible while allowing for a certain margin of error (50). In SVR, the model uses a kernel function to map the training data to a higher-dimensional space, obtaining a regression hyperplane that best fits the training dataset within the boundary lines. The kernel function used in this study is the radial basis function (RBF). To develop the model effectively, an optimization problem was solved using the objective function:

$$\frac{1}{2}\parallel w\parallel^{2}+C\sum_{i=1}^{N} \left( \xi_{i}+\xi_{i}^{*} \right)\to min$$

Where $w$ represents the weight vector of the hyperplane, $C$ is the regularization parameter controlling the complexity of the model, $N$ is the number of training samples, $\xi_{i}$ and $\xi_{i}^{*}$ represent the functional margin between sample points and the hyperplane (52).

The standard SVR equation is:

$$\begin{matrix} y=\sum_{i=1}^{N} (\alpha_{i}^{*}-\alpha)k(x_{i},x)+b \end{matrix}$$

where $y$ is the predicted output, $\alpha_{i}^{*}$ and $\alpha$ are Lagrange multipliers, $k(x_{i},x)$ is the kernel function, and $b$ is the bias term (47).

SVR uses the $\epsilon$-insensitive loss function:

$$L_{\epsilon}(y,y)=\left\{ \begin{matrix} 0 & \text{if}\text{ }|y-y|\leq\epsilon, \\ |y-y|-\epsilon& \text{if}\text{ }\left| y-y \right|>\epsilon. \end{matrix} \right.$$

This loss function ignores small deviations within the margin $\epsilon$, ensuring robustness to minor variations while focusing on larger deviations.

## Random Forest Regression

The core of the random forest (RF) regression (RFR) algorithm lies in a set of regression models based on decision trees (DT), commonly referred to as "base learners". RFR achieves parallel processing of data by simultaneously employing multiple DTs that are mutually independent (7). During the training process, random sub-samples are drawn from the original training set using bootstrap sampling techniques. Once the model training is completed, the predictions are aggregated by computing the average of the predicted values of each DT to generate the combined result. This combination of techniques is also known as "bagging". By utilizing bagging for training, RFR improves its generalization ability by reducing the sensitivity of the model to changes in data (13). In the framework of RF, the objective is to construct a prediction function $f(x)$, which accurately estimates the corresponding response value $y$ given a set of random input variables $x=[x_{1},x_{2},...,x_{m}]$:

$$y=\frac{1}{J}\sum_{j=1}^{J} h_{j}\left( x \right)$$

Where $J$ is the total number of DTs in the RFR, $h_{j}\left( x \right)$ is the prediction of the $J$-th DT in the RFR for input $x$. Each individual tree in RFR minimizes the MSE during the training phase.

## Light Gradient Boosting Machine for Regression

Light gradient boosting machine (LightGBM) is a high-performance gradient boosting decision tree framework specifically optimized for large-scale datasets. This algorithm utilizes a histogram-based approach to enhance computational efficiency and reduce memory consumption. Additionally, advanced techniques such as gradient-based one-side sampling (GOSS) and exclusive feature bundling (EFB) further optimize the training process: GOSS enhances information gain by prioritizing data with larger gradients, while EFB consolidates highly correlated features to streamline computation. By adopting a leaf-wise growth strategy, LightGBM effectively reduces overfitting in complex datasets and achieves superior accuracy and scalability, offering a versatile and efficient solution for regression tasks (7,39). LightGBM builds DTs iteratively, with each tree minimizing residual errors from the previous iteration:

$$y=\sum_{t=1}^{T} f_{t}(x)$$

where $T$is the total number of trees, $f_{t}(x)$ is the prediction result of a single DT. Like RFR, LightGBM iteratively builds DTs to minimize the MSE, effectively reducing prediction errors in regression tasks.
