# Supplementary figures and images for: Machine Learning in Rugby Union: Predicting and Identifying Key Performance Indicators for Professional Rugby Union Players in Match Play Based Workload
Source: Eur J Sport Sci. 2025 Aug 22;25(9):e70042. doi: 10.1002/ejsc.70042 (PMC12373978; doi:10.1002/ejsc.70042)

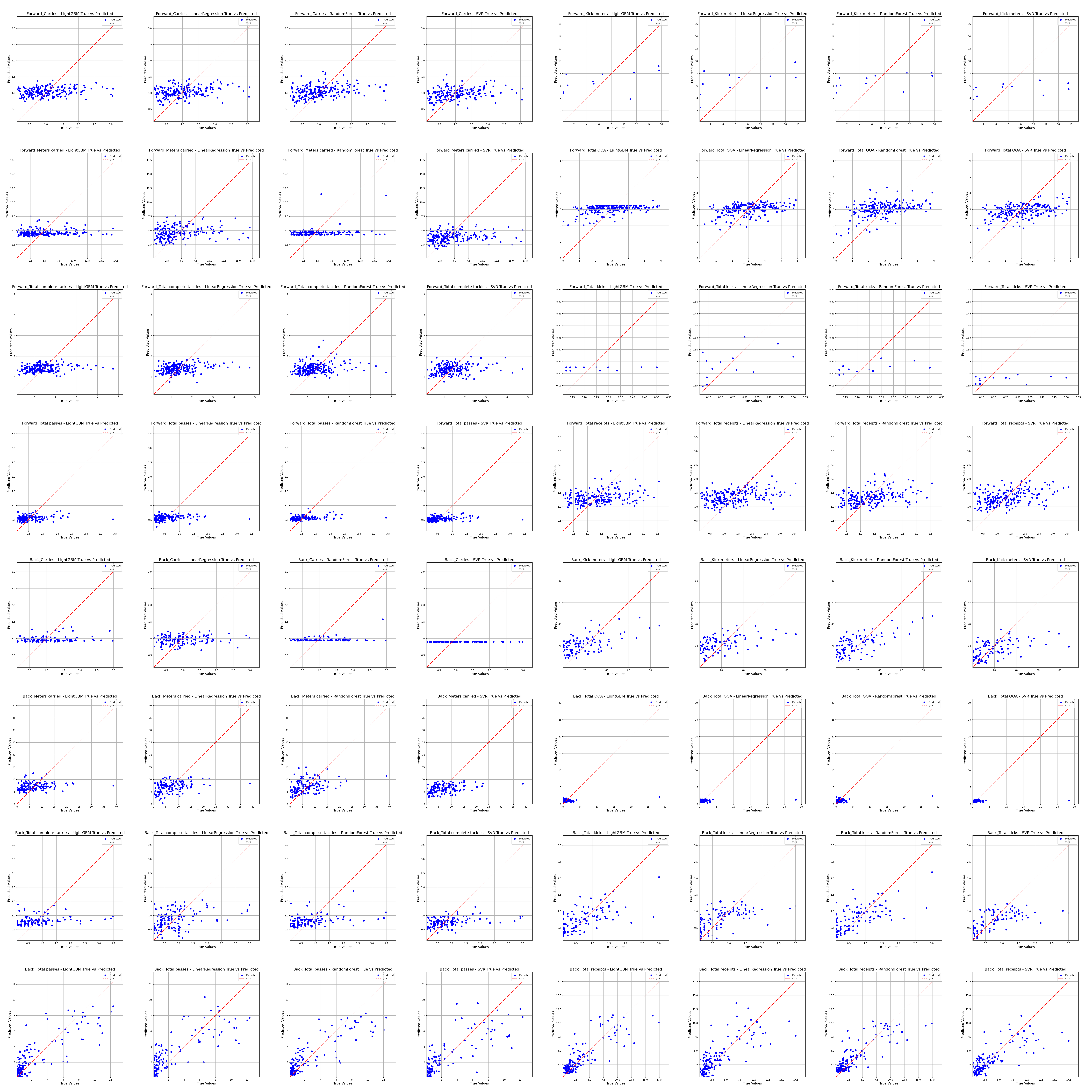

Supplement: Supplementary file 2 — Figure S1: Comparison of predictive performance of different machine learning models: separate presentation of model results. [file EJSC-25-e70042-s004.png]

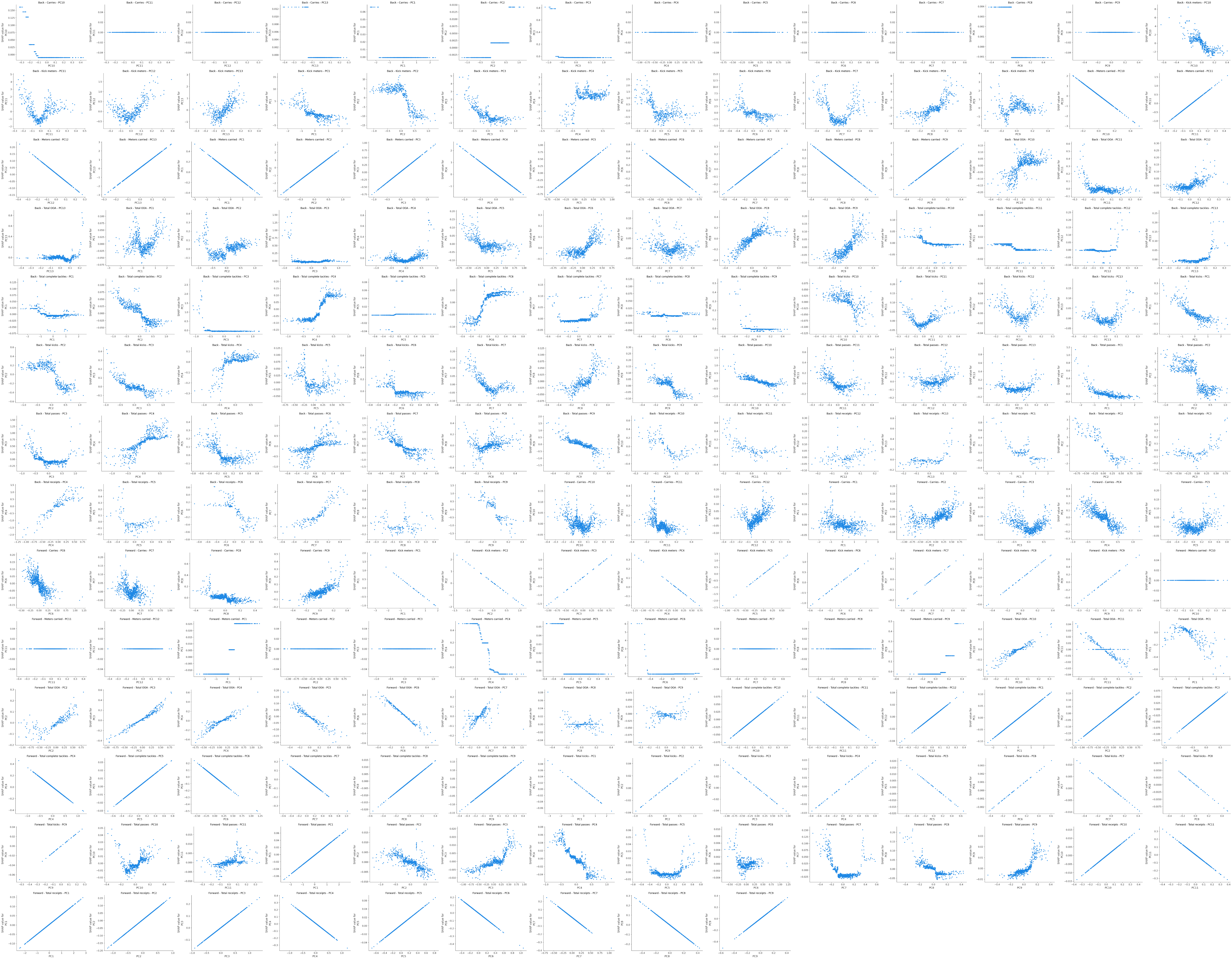

Supplement: Supplementary file 3 — Figure S2: Univariate partial dependence plots for each principal component. [file EJSC-25-e70042-s006.png]
