# Supplementary material for: Machine Learning in Rugby Union: Predicting and Identifying Key Performance Indicators for Professional Rugby Union Players in Match Play Based Workload
Source: Eur J Sport Sci. 2025 Aug 22;25(9):e70042. doi: 10.1002/ejsc.70042 (PMC12373978; doi:10.1002/ejsc.70042)
Supplement: Supplementary file 4 — Table S1: Correlation coefficients between single features and KPI labels for forwards and backs. [file EJSC-25-e70042-s003.pdf]

KPI	Feature	Forward<br>***R***	Back<br>***R***
	Load_7_Days_TD	-0.07	-0.08
	Load_14_Days_TD	-0.07	-0.08
	Load_21_Days_TD	-0.06	-0.07
	Load_7_Days_PL	-0.08	-0.07
	Load_14_Days_PL	-0.07	-0.06
	Load_21_Days_PL	-0.05	-0.05
	Load_7_Days_MSR	0.01	-0.09
	Load_14_Days_MSR	-0.01	-0.08
	Load_21_Days_MSR	-0.01	-0.07
	Load_7_Days_HSR	0.05	-0.04
	Load_14_Days_HSR	0.02	-0.04
	Load_21_Days_HSR	0.02	-0.04
	Load_7_Days_VHSR	0.04	-0.01
	Load_14_Days_VHSR	0.03	-0.03
	Load_21_Days_VHSR	0.03	-0.03
	Load_7_Days_SR	0.01	0.00
	Load_14_Days_SR	0.00	-0.03
	Load_21_Days_SR	-0.01	-0.03
	Load_7_Days_AZ1	-0.04	-0.09
	Load_14_Days_AZ1	-0.05	-0.10
	Load_21_Days_AZ1	-0.03	-0.08
	Load_7_Days_AZ2	-0.01	-0.08
	Load_14_Days_AZ2	-0.02	-0.07
	Load_21_Days_AZ2	-0.01	-0.07
	Load_7_Days_AZ3	0.05	-0.06
	Load_14_Days_AZ3	0.04	-0.05
Carries	Load_21_Days_AZ3	0.03	-0.05
	Load_7_Days_DZ1	-0.02	-0.06
	Load_14_Days_DZ1	-0.02	-0.06
	Load_21_Days_DZ1	-0.01	-0.06
	Load_7_Days_DZ2	-0.01	-0.04
	Load_14_Days_DZ2	0.01	-0.06
	Load_21_Days_DZ2	0.01	-0.07
	Load_7_Days_DZ3	0.09	-0.02
	Load_14_Days_DZ3	0.09	-0.01
	Load_21_Days_DZ3	0.09	-0.01
	Load_7_Days_ADZ1	-0.01	-0.06
	Load_14_Days_ADZ1	-0.01	-0.07
	Load_21_Days_ADZ1	0.00	-0.06
	Load_7_Days_ADZ2	0.00	-0.09
	Load_14_Days_ADZ2	-0.01	-0.08
	Load_21_Days_ADZ2	-0.01	-0.08
	Load_7_Days_ADZ3	0.02	-0.07
	Load_14_Days_ADZ3	0.01	-0.05
	Load_21_Days_ADZ3	0.00	-0.06
	Load_7_Days_RHIE	0.07	-0.01
	Load_14_Days_RHIE	0.08	-0.01
	Load_21_Days_RHIE	0.08	-0.01
	Load_7_Days_Contact involvement total count	0.02	0.01
	Load_14_Days_Contact involvement total count	0.05	0.01
	Load_21_Days_Contact involvement total count	0.06	0.02
	Load_7_Days_Heart rate exertion	-0.08	-0.03
	Load_14_Days_Heart rate exertion	-0.09	-0.04
	Load_21_Days_Heart rate exertion	-0.08	-0.03

	Load_7_Days_TD	-0.16	-0.15
	Load_14_Days_TD	-0.15	-0.09
	Load_21_Days_TD	-0.35	-0.09
	Load_7_Days_PL	-0.17	-0.13
	Load_14_Days_PL	-0.15	-0.07
	Load_21_Days_PL	-0.35	-0.06
	Load_7_Days_MSR	-0.21	-0.06
	Load_14_Days_MSR	-0.15	0.00
	Load_21_Days_MSR	-0.33	0.01
	Load_7_Days_HSR	-0.23	-0.15
	Load_14_Days_HSR	-0.18	-0.12
	Load_21_Days_HSR	-0.29	-0.13
	Load_7_Days_VHSR	-0.11	-0.29
	Load_14_Days_VHSR	-0.08	-0.30
	Load_21_Days_VHSR	-0.18	-0.32
	Load_7_Days_SR	0.11	-0.34
	Load_14_Days_SR	0.12	-0.35
	Load_21_Days_SR	-0.02	-0.36
	Load_7_Days_AZ1	-0.28	-0.07
	Load_14_Days_AZ1	-0.25	-0.04
	Load_21_Days_AZ1	-0.39	-0.03
	Load_7_Days_AZ2	-0.27	-0.14
	Load_14_Days_AZ2	-0.20	-0.09
	Load_21_Days_AZ2	-0.37	-0.08
	Load_7_Days_AZ3	-0.09	-0.15
	Load_14_Days_AZ3	-0.07	-0.14
Kick meters	Load_21_Days_AZ3	-0.15	-0.14
	Load_7_Days_DZ1	-0.21	-0.07
	Load_14_Days_DZ1	-0.15	-0.02
	Load_21_Days_DZ1	-0.32	0.00
	Load_7_Days_DZ2	-0.33	-0.07
	Load_14_Days_DZ2	-0.26	-0.05
	Load_21_Days_DZ2	-0.36	-0.04
	Load_7_Days_DZ3	-0.07	-0.15
	Load_14_Days_DZ3	-0.04	-0.14
	Load_21_Days_DZ3	-0.17	-0.12
	Load_7_Days_ADZ1	-0.24	-0.17
	Load_14_Days_ADZ1	-0.27	-0.15
	Load_21_Days_ADZ1	-0.43	-0.13
	Load_7_Days_ADZ2	-0.14	-0.20
	Load_14_Days_ADZ2	-0.11	-0.17
	Load_21_Days_ADZ2	-0.27	-0.16
	Load_7_Days_ADZ3	-0.06	-0.17
	Load_14_Days_ADZ3	-0.03	-0.17
	Load_21_Days_ADZ3	-0.12	-0.18
	Load_7_Days_RHIE	-0.16	-0.22
	Load_14_Days_RHIE	-0.15	-0.19
	Load_21_Days_RHIE	-0.24	-0.19
	Load_7_Days_Contact involvement total count	-0.10	-0.18
	Load_14_Days_Contact involvement total count	-0.08	-0.15
	Load_21_Days_Contact involvement total count	-0.19	-0.17
	Load_7_Days_Heart rate exertion	-0.15	-0.09
	Load_14_Days_Heart rate exertion	-0.14	-0.05
	Load_21_Days_Heart rate exertion	-0.34	-0.05
	Load_7_Days_TD	-0.01	0.01
	Load_14_Days_TD	-0.02	-0.05

	Load_21_Days_TD	-0.04	-0.11
	Load_7_Days_PL	-0.02	-0.04
	Load_14_Days_PL	-0.02	-0.09
	Load_21_Days_PL	-0.03	-0.13
	Load_7_Days_MSR	0.07	-0.12
	Load_14_Days_MSR	0.05	-0.18
	Load_21_Days_MSR	0.04	-0.22
	Load_7_Days_HSR	0.11	0.02
	Load_14_Days_HSR	0.09	-0.05
	Load_21_Days_HSR	0.09	-0.09
	Load_7_Days_VHSR	0.11	0.16
	Load_14_Days_VHSR	0.10	0.11
	Load_21_Days_VHSR	0.10	0.09
	Load_7_Days_SR	0.08	0.18
	Load_14_Days_SR	0.05	0.15
	Load_21_Days_SR	0.06	0.15
	Load_7_Days_AZ1	0.00	-0.07
	Load_14_Days_AZ1	-0.01	-0.13
	Load_21_Days_AZ1	-0.02	-0.18
	Load_7_Days_AZ2	0.03	-0.04
	Load_14_Days_AZ2	0.02	-0.07
	Load_21_Days_AZ2	0.01	-0.11
	Load_7_Days_AZ3	0.11	0.06
	Load_14_Days_AZ3	0.11	0.02
Meters carried	Load_21_Days_AZ3	0.10	0.01
	Load_7_Days_DZ1	0.01	-0.07
	Load_14_Days_DZ1	0.01	-0.10
	Load_21_Days_DZ1	0.00	-0.15
	Load_7_Days_DZ2	0.04	0.00
	Load_14_Days_DZ2	0.05	-0.08
	Load_21_Days_DZ2	0.04	-0.11
	Load_7_Days_DZ3	0.16	0.11
	Load_14_Days_DZ3	0.15	0.07
	Load_21_Days_DZ3	0.14	0.04
	Load_7_Days_ADZ1	0.05	0.03
	Load_14_Days_ADZ1	0.06	-0.02
	Load_21_Days_ADZ1	0.05	-0.06
	Load_7_Days_ADZ2	0.06	0.02
	Load_14_Days_ADZ2	0.06	-0.02
	Load_21_Days_ADZ2	0.04	-0.04
	Load_7_Days_ADZ3	0.09	0.05
	Load_14_Days_ADZ3	0.09	0.01
	Load_21_Days_ADZ3	0.07	0.00
	Load_7_Days_RHIE	0.14	0.10
	Load_14_Days_RHIE	0.13	0.05
	Load_21_Days_RHIE	0.12	0.02
	Load_7_Days_Contact involvement total count	0.02	0.05
	Load_14_Days_Contact involvement total count	0.03	0.04
	Load_21_Days_Contact involvement total count	0.03	0.04
	Load_7_Days_Heart rate exertion	-0.02	0.05
	Load_14_Days_Heart rate exertion	-0.04	-0.01
	Load_21_Days_Heart rate exertion	-0.06	-0.07
	Load_7_Days_TD	-0.06	-0.10
	Load_14_Days_TD	-0.01	-0.04
	Load_21_Days_TD	0.02	-0.01
	Load_7_Days_PL	-0.09	-0.06

	Load_14_Days_PL	-0.04	0.00
	Load_21_Days_PL	-0.02	0.03
	Load_7_Days_MSR	-0.01	0.00
	Load_14_Days_MSR	0.02	0.08
	Load_21_Days_MSR	0.04	0.10
	Load_7_Days_HSR	-0.02	-0.08
	Load_14_Days_HSR	0.02	0.01
	Load_21_Days_HSR	0.03	0.02
	Load_7_Days_VHSR	-0.01	-0.16
	Load_14_Days_VHSR	0.03	-0.12
	Load_21_Days_VHSR	0.03	-0.13
	Load_7_Days_SR	-0.01	-0.15
	Load_14_Days_SR	0.02	-0.15
	Load_21_Days_SR	0.03	-0.16
	Load_7_Days_AZ1	-0.02	-0.04
	Load_14_Days_AZ1	-0.01	0.02
	Load_21_Days_AZ1	0.01	0.06
	Load_7_Days_AZ2	-0.03	-0.03
	Load_14_Days_AZ2	0.01	0.01
	Load_21_Days_AZ2	0.03	0.02
	Load_7_Days_AZ3	0.00	-0.10
	Load_14_Days_AZ3	0.05	-0.06
	Load_21_Days_AZ3	0.07	-0.06
Total complete tackles	Load_7_Days_DZ1	-0.02	-0.01
	Load_14_Days_DZ1	0.00	0.03
	Load_21_Days_DZ1	0.03	0.06
	Load_7_Days_DZ2	0.03	-0.05
	Load_14_Days_DZ2	0.04	0.00
	Load_21_Days_DZ2	0.06	0.00
	Load_7_Days_DZ3	0.05	-0.13
	Load_14_Days_DZ3	0.08	-0.09
	Load_21_Days_DZ3	0.09	-0.08
	Load_7_Days_ADZ1	-0.04	-0.09
	Load_14_Days_ADZ1	-0.02	-0.04
	Load_21_Days_ADZ1	0.01	-0.02
	Load_7_Days_ADZ2	-0.05	-0.10
	Load_14_Days_ADZ2	0.00	-0.07
	Load_21_Days_ADZ2	0.02	-0.05
	Load_7_Days_ADZ3	-0.02	-0.10
	Load_14_Days_ADZ3	0.04	-0.05
	Load_21_Days_ADZ3	0.05	-0.06
	Load_7_Days_RHIE	0.03	-0.10
	Load_14_Days_RHIE	0.07	-0.05
	Load_21_Days_RHIE	0.08	-0.04
	Load_7_Days_Contact involvement total count	0.05	0.03
	Load_14_Days_Contact involvement total count	0.06	0.02
	Load_21_Days_Contact involvement total count	0.07	0.04
	Load_7_Days_Heart rate exertion	-0.13	-0.10
	Load_14_Days_Heart rate exertion	-0.07	-0.06
	Load_21_Days_Heart rate exertion	-0.05	-0.03
	Load_7_Days_TD	-0.23	-0.13
	Load_14_Days_TD	-0.29	-0.11
	Load_21_Days_TD	-0.36	-0.09
	Load_7_Days_PL	-0.21	-0.08
	Load_14_Days_PL	-0.27	-0.06
	Load_21_Days_PL	-0.32	-0.03

	Load_7_Days_MSR	-0.15	0.03
	Load_14_Days_MSR	-0.23	0.07
	Load_21_Days_MSR	-0.29	0.10
	Load_7_Days_HSR	-0.23	-0.11
	Load_14_Days_HSR	-0.27	-0.10
	Load_21_Days_HSR	-0.31	-0.10
	Load_7_Days_VHSR	-0.17	-0.32
	Load_14_Days_VHSR	-0.20	-0.35
	Load_21_Days_VHSR	-0.26	-0.36
	Load_7_Days_SR	-0.06	-0.40
	Load_14_Days_SR	-0.06	-0.41
	Load_21_Days_SR	-0.13	-0.41
	Load_7_Days_AZ1	-0.20	-0.01
	Load_14_Days_AZ1	-0.28	0.00
	Load_21_Days_AZ1	-0.35	0.02
	Load_7_Days_AZ2	-0.19	-0.12
	Load_14_Days_AZ2	-0.27	-0.09
	Load_21_Days_AZ2	-0.32	-0.07
	Load_7_Days_AZ3	-0.10	-0.19
	Load_14_Days_AZ3	-0.20	-0.20
Total kicks	Load_21_Days_AZ3	-0.18	-0.19
	Load_7_Days_DZ1	-0.20	0.01
	Load_14_Days_DZ1	-0.25	0.03
	Load_21_Days_DZ1	-0.29	0.06
	Load_7_Days_DZ2	-0.21	-0.04
	Load_14_Days_DZ2	-0.27	-0.04
	Load_21_Days_DZ2	-0.31	-0.02
	Load_7_Days_DZ3	-0.08	-0.18
	Load_14_Days_DZ3	-0.15	-0.19
	Load_21_Days_DZ3	-0.18	-0.16
	Load_7_Days_ADZ1	-0.15	-0.16
	Load_14_Days_ADZ1	-0.29	-0.16
	Load_21_Days_ADZ1	-0.36	-0.14
	Load_7_Days_ADZ2	-0.15	-0.22
	Load_14_Days_ADZ2	-0.24	-0.21
	Load_21_Days_ADZ2	-0.29	-0.19
	Load_7_Days_ADZ3	-0.09	-0.21
	Load_14_Days_ADZ3	-0.15	-0.21
	Load_21_Days_ADZ3	-0.17	-0.21
	Load_7_Days_RHIE	-0.18	-0.22
	Load_14_Days_RHIE	-0.24	-0.21
	Load_21_Days_RHIE	-0.25	-0.20
	Load_7_Days_Contact involvement total count	-0.09	-0.18
	Load_14_Days_Contact involvement total count	-0.07	-0.18
	Load_21_Days_Contact involvement total count	-0.13	-0.20
	Load_7_Days_Heart rate exertion	-0.22	-0.10
	Load_14_Days_Heart rate exertion	-0.28	-0.09
	Load_21_Days_Heart rate exertion	-0.36	-0.08
	Load_7_Days_TD	-0.09	-0.01
	Load_14_Days_TD	-0.08	-0.08
	Load_21_Days_TD	-0.03	-0.05
	Load_7_Days_PL	-0.07	-0.01
	Load_14_Days_PL	-0.04	-0.06
	Load_21_Days_PL	0.01	-0.03
	Load_7_Days_MSR	-0.07	0.01
	Load_14_Days_MSR	-0.07	-0.04

Total OOA	Load_21_Days_MSR	-0.04	-0.02
	Load_7_Days_HSR	-0.13	0.00
	Load_14_Days_HSR	-0.14	-0.03
	Load_21_Days_HSR	-0.13	-0.02
	Load_7_Days_VHSR	-0.18	-0.04
	Load_14_Days_VHSR	-0.19	-0.05
	Load_21_Days_VHSR	-0.18	-0.04
	Load_7_Days_SR	-0.18	-0.08
	Load_14_Days_SR	-0.20	-0.08
	Load_21_Days_SR	-0.19	-0.07
	Load_7_Days_AZ1	-0.06	0.03
	Load_14_Days_AZ1	-0.06	-0.03
	Load_21_Days_AZ1	-0.01	0.00
	Load_7_Days_AZ2	-0.10	0.08
	Load_14_Days_AZ2	-0.10	0.02
	Load_21_Days_AZ2	-0.05	0.04
	Load_7_Days_AZ3	-0.10	-0.02
	Load_14_Days_AZ3	-0.12	-0.04
	Load_21_Days_AZ3	-0.10	-0.02
	Load_7_Days_DZ1	-0.07	0.07
	Load_14_Days_DZ1	-0.06	-0.01
	Load_21_Days_DZ1	-0.01	0.01
	Load_7_Days_DZ2	-0.09	0.02
	Load_14_Days_DZ2	-0.08	-0.02
	Load_21_Days_DZ2	-0.04	-0.02
	Load_7_Days_DZ3	-0.13	-0.05
	Load_14_Days_DZ3	-0.12	-0.06
	Load_21_Days_DZ3	-0.11	-0.06
	Load_7_Days_ADZ1	-0.10	0.01
	Load_14_Days_ADZ1	-0.11	-0.03
	Load_21_Days_ADZ1	-0.07	0.00
	Load_7_Days_ADZ2	-0.12	0.05
	Load_14_Days_ADZ2	-0.12	0.00
	Load_21_Days_ADZ2	-0.08	0.01
	Load_7_Days_ADZ3	-0.12	0.00
	Load_14_Days_ADZ3	-0.12	-0.02
	Load_21_Days_ADZ3	-0.10	-0.01
	Load_7_Days_RHIE	-0.16	-0.03
	Load_14_Days_RHIE	-0.15	-0.06
	Load_21_Days_RHIE	-0.13	-0.04
	Load_7_Days_Contact involvement total count	-0.06	0.11
	Load_14_Days_Contact involvement total count	-0.05	0.07
	Load_21_Days_Contact involvement total count	-0.03	0.08
	Load_7_Days_Heart rate exertion	-0.06	-0.02
	Load_14_Days_Heart rate exertion	-0.05	-0.08
	Load_21_Days_Heart rate exertion	0.00	-0.05
	Load_7_Days_TD	0.04	0.02
	Load_14_Days_TD	0.02	0.02
	Load_21_Days_TD	-0.01	0.03
	Load_7_Days_PL	0.04	0.13
	Load_14_Days_PL	0.02	0.16
	Load_21_Days_PL	0.00	0.17
	Load_7_Days_MSR	0.11	0.33
	Load_14_Days_MSR	0.09	0.35
	Load_21_Days_MSR	0.07	0.36
	Load_7_Days_HSR	0.10	0.09

	Load_14_Days_HSR	0.10	0.11
	Load_21_Days_HSR	0.08	0.10
	Load_7_Days_VHSR	0.11	-0.28
	Load_14_Days_VHSR	0.12	-0.29
	Load_21_Days_VHSR	0.11	-0.30
	Load_7_Days_SR	0.08	-0.40
	Load_14_Days_SR	0.08	-0.40
	Load_21_Days_SR	0.08	-0.41
	Load_7_Days_AZ1	0.07	0.18
	Load_14_Days_AZ1	0.04	0.21
	Load_21_Days_AZ1	0.02	0.24
	Load_7_Days_AZ2	0.07	-0.03
	Load_14_Days_AZ2	0.04	-0.04
	Load_21_Days_AZ2	0.02	-0.04
	Load_7_Days_AZ3	0.08	-0.27
	Load_14_Days_AZ3	0.06	-0.28
Total passes	Load_21_Days_AZ3	0.04	-0.28
	Load_7_Days_DZ1	0.05	0.21
	Load_14_Days_DZ1	0.02	0.21
	Load_21_Days_DZ1	0.00	0.24
	Load_7_Days_DZ2	0.09	0.08
	Load_14_Days_DZ2	0.06	0.09
	Load_21_Days_DZ2	0.04	0.09
	Load_7_Days_DZ3	0.14	-0.17
	Load_14_Days_DZ3	0.12	-0.18
	Load_21_Days_DZ3	0.10	-0.18
	Load_7_Days_ADZ1	0.07	-0.05
	Load_14_Days_ADZ1	0.06	-0.04
	Load_21_Days_ADZ1	0.04	-0.04
	Load_7_Days_ADZ2	0.08	-0.21
	Load_14_Days_ADZ2	0.06	-0.23
	Load_21_Days_ADZ2	0.03	-0.22
	Load_7_Days_ADZ3	0.07	-0.27
	Load_14_Days_ADZ3	0.05	-0.29
	Load_21_Days_ADZ3	0.03	-0.28
	Load_7_Days_RHIE	0.15	-0.14
	Load_14_Days_RHIE	0.12	-0.14
	Load_21_Days_RHIE	0.11	-0.15
	Load_7_Days_Contact involvement total count	0.06	-0.18
	Load_14_Days_Contact involvement total count	0.06	-0.18
	Load_21_Days_Contact involvement total count	0.06	-0.18
	Load_7_Days_Heart rate exertion	-0.02	0.00
	Load_14_Days_Heart rate exertion	-0.05	-0.01
	Load_21_Days_Heart rate exertion	-0.08	0.00
	Load_7_Days_TD	-0.05	0.01
	Load_14_Days_TD	-0.04	0.01
	Load_21_Days_TD	-0.04	0.02
	Load_7_Days_PL	-0.05	0.12
	Load_14_Days_PL	-0.03	0.13
	Load_21_Days_PL	-0.02	0.15
	Load_7_Days_MSR	0.06	0.32
	Load_14_Days_MSR	0.04	0.33
	Load_21_Days_MSR	0.03	0.35
	Load_7_Days_HSR	0.11	0.09
	Load_14_Days_HSR	0.08	0.09
	Load_21_Days_HSR	0.07	0.09

	Load_7_Days_VHSR	0.10	-0.28
	Load_14_Days_VHSR	0.09	-0.30
	Load_21_Days_VHSR	0.08	-0.30
	Load_7_Days_SR	0.04	-0.39
	Load_14_Days_SR	0.03	-0.40
	Load_21_Days_SR	0.02	-0.41
	Load_7_Days_AZ1	0.00	0.18
	Load_14_Days_AZ1	0.00	0.19
	Load_21_Days_AZ1	0.01	0.22
	Load_7_Days_AZ2	0.03	-0.03
	Load_14_Days_AZ2	0.03	-0.05
	Load_21_Days_AZ2	0.03	-0.04
	Load_7_Days_AZ3	0.09	-0.26
	Load_14_Days_AZ3	0.07	-0.28
Total receipts	Load_21_Days_AZ3	0.06	-0.27
	Load_7_Days_DZ1	0.00	0.22
	Load_14_Days_DZ1	0.01	0.21
	Load_21_Days_DZ1	0.01	0.25
	Load_7_Days_DZ2	0.04	0.10
	Load_14_Days_DZ2	0.04	0.09
	Load_21_Days_DZ2	0.04	0.10
	Load_7_Days_DZ3	0.14	-0.15
	Load_14_Days_DZ3	0.14	-0.18
	Load_21_Days_DZ3	0.14	-0.17
	Load_7_Days_ADZ1	0.04	-0.05
	Load_14_Days_ADZ1	0.05	-0.05
	Load_21_Days_ADZ1	0.05	-0.04
	Load_7_Days_ADZ2	0.05	-0.21
	Load_14_Days_ADZ2	0.04	-0.23
	Load_21_Days_ADZ2	0.04	-0.22
	Load_7_Days_ADZ3	0.06	-0.26
	Load_14_Days_ADZ3	0.05	-0.28
	Load_21_Days_ADZ3	0.03	-0.28
	Load_7_Days_RHIE	0.14	-0.14
	Load_14_Days_RHIE	0.14	-0.15
	Load_21_Days_RHIE	0.13	-0.15
	Load_7_Days_Contact involvement total count	0.06	-0.19
	Load_14_Days_Contact involvement total count	0.08	-0.19
	Load_21_Days_Contact involvement total count	0.09	-0.19
	Load_7_Days_Heart rate exertion	-0.08	0.00
	Load_14_Days_Heart rate exertion	-0.07	-0.02
	Load_21_Days_Heart rate exertion	-0.08	0.00
