# Supplementary material for: Machine Learning in Rugby Union: Predicting and Identifying Key Performance Indicators for Professional Rugby Union Players in Match Play Based Workload
Source: Eur J Sport Sci. 2025 Aug 22;25(9):e70042. doi: 10.1002/ejsc.70042 (PMC12373978; doi:10.1002/ejsc.70042)
Supplement: Supplementary file 5 — Table S2: The variance ratio of each principal component. [file EJSC-25-e70042-s005.pdf]

Position_KPI	Principal Component	Variance Ratio
Forward_Carries	PC1	0.55
Forward_Carries	PC2	0.11
Forward_Carries	PC3	0.09
Forward_Carries	PC4	0.08
Forward_Carries	PC5	0.04
Forward_Carries	PC6	0.02
Forward_Carries	PC7	0.02
Forward_Carries	PC8	0.01
Forward_Carries	PC9	0.01
Forward_Carries	PC10	0.01
Forward_Carries	PC11	0.01
Forward_Carries	PC12	0.01
Forward_Kick meters	PC1	0.48
Forward_Kick meters	PC2	0.15
Forward_Kick meters	PC3	0.10
Forward_Kick meters	PC4	0.08
Forward_Kick meters	PC5	0.05
Forward_Kick meters	PC6	0.04
Forward_Kick meters	PC7	0.03
Forward_Kick meters	PC8	0.02
Forward_Kick meters	PC9	0.01
Forward_Meters carried	PC1	0.56
Forward_Meters carried	PC2	0.11
Forward_Meters carried	PC3	0.09
Forward_Meters carried	PC4	0.08
Forward_Meters carried	PC5	0.04
Forward_Meters carried	PC6	0.02
Forward_Meters carried	PC7	0.02
Forward_Meters carried	PC8	0.01
Forward_Meters carried	PC9	0.01
Forward_Meters carried	PC10	0.01
Forward_Meters carried	PC11	0.01
Forward_Meters carried	PC12	0.01
Forward_Total complete tackles	PC1	0.56
Forward_Total complete tackles	PC2	0.11
Forward_Total complete tackles	PC3	0.09
Forward_Total complete tackles	PC4	0.08
Forward_Total complete tackles	PC5	0.04
Forward_Total complete tackles	PC6	0.02
Forward_Total complete tackles	PC7	0.02
Forward_Total complete tackles	PC8	0.01
Forward_Total complete tackles	PC9	0.01
Forward_Total complete tackles	PC10	0.01
Forward_Total complete tackles	PC11	0.01
Forward_Total complete tackles	PC12	0.01
Forward_Total kicks	PC1	0.54
Forward_Total kicks	PC2	0.11
Forward_Total kicks	PC3	0.11
Forward_Total kicks	PC4	0.08
Forward_Total kicks	PC5	0.04
Forward_Total kicks	PC6	0.03
Forward_Total kicks	PC7	0.02
Forward_Total kicks	PC8	0.02

Forward_Total kicks	PC9	0.01
Forward_Total OOA	PC1	0.55
Forward_Total OOA	PC2	0.11
Forward_Total OOA	PC3	0.09
Forward_Total OOA	PC4	0.08
Forward_Total OOA	PC5	0.04
Forward_Total OOA	PC6	0.02
Forward_Total OOA	PC7	0.02
Forward_Total OOA	PC8	0.01
Forward_Total OOA	PC9	0.01
Forward_Total OOA	PC10	0.01
Forward_Total OOA	PC11	0.01
Forward_Total passes	PC1	0.57
Forward_Total passes	PC2	0.10
Forward_Total passes	PC3	0.09
Forward_Total passes	PC4	0.07
Forward_Total passes	PC5	0.04
Forward_Total passes	PC6	0.02
Forward_Total passes	PC7	0.02
Forward_Total passes	PC8	0.01
Forward_Total passes	PC9	0.01
Forward_Total passes	PC10	0.01
Forward_Total passes	PC11	0.01
Forward_Total receipts	PC1	0.56
Forward_Total receipts	PC2	0.11
Forward_Total receipts	PC3	0.09
Forward_Total receipts	PC4	0.07
Forward_Total receipts	PC5	0.04
Forward_Total receipts	PC6	0.02
Forward_Total receipts	PC7	0.02
Forward_Total receipts	PC8	0.01
Forward_Total receipts	PC9	0.01
Forward_Total receipts	PC10	0.01
Forward_Total receipts	PC11	0.01
Back_Carries	PC1	0.44
Back_Carries	PC2	0.13
Back_Carries	PC3	0.11
Back_Carries	PC4	0.09
Back_Carries	PC5	0.05
Back_Carries	PC6	0.04
Back_Carries	PC7	0.02
Back_Carries	PC8	0.02
Back_Carries	PC9	0.01
Back_Carries	PC10	0.01
Back_Carries	PC11	0.01
Back_Carries	PC12	0.01
Back_Carries	PC13	0.01
Back_Kick meters	PC1	0.45
Back_Kick meters	PC2	0.13
Back_Kick meters	PC3	0.10
Back_Kick meters	PC4	0.08
Back_Kick meters	PC5	0.06
Back_Kick meters	PC6	0.04
Back_Kick meters	PC7	0.03

Back_Kick meters	PC8	0.02
Back_Kick meters	PC9	0.01
Back_Kick meters	PC10	0.01
Back_Kick meters	PC11	0.01
Back_Kick meters	PC12	0.01
Back_Kick meters	PC13	0.01
Back_Meters carried	PC1	0.44
Back_Meters carried	PC2	0.13
Back_Meters carried	PC3	0.11
Back_Meters carried	PC4	0.09
Back_Meters carried	PC5	0.05
Back_Meters carried	PC6	0.04
Back_Meters carried	PC7	0.02
Back_Meters carried	PC8	0.02
Back_Meters carried	PC9	0.01
Back_Meters carried	PC10	0.01
Back_Meters carried	PC11	0.01
Back_Meters carried	PC12	0.01
Back_Meters carried	PC13	0.01
Back_Total complete tackles	PC1	0.44
Back_Total complete tackles	PC2	0.13
Back_Total complete tackles	PC3	0.10
Back_Total complete tackles	PC4	0.09
Back_Total complete tackles	PC5	0.06
Back_Total complete tackles	PC6	0.04
Back_Total complete tackles	PC7	0.03
Back_Total complete tackles	PC8	0.02
Back_Total complete tackles	PC9	0.01
Back_Total complete tackles	PC10	0.01
Back_Total complete tackles	PC11	0.01
Back_Total complete tackles	PC12	0.01
Back_Total complete tackles	PC13	0.01
Back_Total kicks	PC1	0.44
Back_Total kicks	PC2	0.14
Back_Total kicks	PC3	0.11
Back_Total kicks	PC4	0.08
Back_Total kicks	PC5	0.06
Back_Total kicks	PC6	0.05
Back_Total kicks	PC7	0.03
Back_Total kicks	PC8	0.02
Back_Total kicks	PC9	0.01
Back_Total kicks	PC10	0.01
Back_Total kicks	PC11	0.01
Back_Total kicks	PC12	0.01
Back_Total kicks	PC13	0.01
Back_Total OOA	PC1	0.43
Back_Total OOA	PC2	0.13
Back_Total OOA	PC3	0.11
Back_Total OOA	PC4	0.09
Back_Total OOA	PC5	0.06
Back_Total OOA	PC6	0.05
Back_Total OOA	PC7	0.03
Back_Total OOA	PC8	0.02
Back_Total OOA	PC9	0.01

Back_Total OOA	PC10	0.01
Back_Total OOA	PC11	0.01
Back_Total OOA	PC12	0.01
Back_Total OOA	PC13	0.01
Back_Total passes	PC1	0.44
Back_Total passes	PC2	0.12
Back_Total passes	PC3	0.11
Back_Total passes	PC4	0.10
Back_Total passes	PC5	0.05
Back_Total passes	PC6	0.04
Back_Total passes	PC7	0.02
Back_Total passes	PC8	0.02
Back_Total passes	PC9	0.01
Back_Total passes	PC10	0.01
Back_Total passes	PC11	0.01
Back_Total passes	PC12	0.01
Back_Total passes	PC13	0.01
Back_Total receipts	PC1	0.42
Back_Total receipts	PC2	0.13
Back_Total receipts	PC3	0.11
Back_Total receipts	PC4	0.09
Back_Total receipts	PC5	0.06
Back_Total receipts	PC6	0.05
Back_Total receipts	PC7	0.02
Back_Total receipts	PC8	0.02
Back_Total receipts	PC9	0.01
Back_Total receipts	PC10	0.01
Back_Total receipts	PC11	0.01
Back_Total receipts	PC12	0.01
Back_Total receipts	PC13	0.01
