# Supplementary material for: Machine Learning in Rugby Union: Predicting and Identifying Key Performance Indicators for Professional Rugby Union Players in Match Play Based Workload
Source: Eur J Sport Sci. 2025 Aug 22;25(9):e70042. doi: 10.1002/ejsc.70042 (PMC12373978; doi:10.1002/ejsc.70042)
Supplement: Supplementary file 7 — Table S4: Correlation coefficients between single principal component and KPI labels for forwards and backs. [file EJSC-25-e70042-s007.pdf]

Position_KPI	Principal Component	Pearson Correlation	Absolute *R*
Back_Carries	PC1	-0.067	0.067
Back_Carries	PC2	0.065	0.065
Back_Carries	PC3	-0.022	0.022
Back_Carries	PC4	0.013	0.013
Back_Carries	PC5	0.006	0.006
Back_Carries	PC6	0.000	0.000
Back_Carries	PC7	0.037	0.037
Back_Carries	PC8	-0.060	0.060
Back_Carries	PC9	-0.042	0.042
Back_Carries	PC10	-0.083	0.083
Back_Carries	PC11	-0.012	0.012
Back_Carries	PC12	0.021	0.021
Back_Carries	PC13	-0.059	0.059
Back_Kick meters	PC1	-0.182	0.182
Back_Kick meters	PC2	-0.253	0.253
Back_Kick meters	PC3	-0.062	0.062
Back_Kick meters	PC4	0.056	0.056
Back_Kick meters	PC5	-0.039	0.039
Back_Kick meters	PC6	-0.137	0.137
Back_Kick meters	PC7	-0.043	0.043
Back_Kick meters	PC8	0.129	0.129
Back_Kick meters	PC9	-0.009	0.009
Back_Kick meters	PC10	-0.130	0.130
Back_Kick meters	PC11	0.019	0.019
Back_Kick meters	PC12	0.047	0.047
Back_Kick meters	PC13	-0.039	0.039
Back_Meters carried	PC1	-0.024	0.024
Back_Meters carried	PC2	0.197	0.197
Back_Meters carried	PC3	0.046	0.046
Back_Meters carried	PC4	-0.122	0.122
Back_Meters carried	PC5	0.049	0.049
Back_Meters carried	PC6	-0.037	0.037
Back_Meters carried	PC7	0.017	0.017
Back_Meters carried	PC8	-0.032	0.032
Back_Meters carried	PC9	0.100	0.100
Back_Meters carried	PC10	-0.126	0.126
Back_Meters carried	PC11	0.064	0.064
Back_Meters carried	PC12	-0.008	0.008
Back_Meters carried	PC13	0.155	0.155
Back_Total complete tackles	PC1	-0.050	0.050
Back_Total complete tackles	PC2	-0.076	0.076
Back_Total complete tackles	PC3	-0.087	0.087
Back_Total complete tackles	PC4	0.118	0.118
Back_Total complete tackles	PC5	-0.029	0.029
Back_Total complete tackles	PC6	0.093	0.093
Back_Total complete tackles	PC7	0.052	0.052
Back_Total complete tackles	PC8	0.007	0.007
Back_Total complete tackles	PC9	-0.049	0.049
Back_Total complete tackles	PC10	-0.085	0.085
Back_Total complete tackles	PC11	-0.064	0.064
Back_Total complete tackles	PC12	0.013	0.013

Back_Total complete tackles	PC13	0.100	0.100
Back_Total kicks	PC1	-0.201	0.201
Back_Total kicks	PC2	-0.298	0.298
Back_Total kicks	PC3	-0.122	0.122
Back_Total kicks	PC4	0.120	0.120
Back_Total kicks	PC5	-0.023	0.023
Back_Total kicks	PC6	-0.130	0.130
Back_Total kicks	PC7	-0.115	0.115
Back_Total kicks	PC8	0.098	0.098
Back_Total kicks	PC9	-0.116	0.116
Back_Total kicks	PC10	-0.040	0.040
Back_Total kicks	PC11	0.026	0.026
Back_Total kicks	PC12	-0.029	0.029
Back_Total kicks	PC13	-0.040	0.040
Back_Total OOA	PC1	0.015	0.015
Back_Total OOA	PC2	0.004	0.004
Back_Total OOA	PC3	-0.056	0.056
Back_Total OOA	PC4	0.092	0.092
Back_Total OOA	PC5	-0.077	0.077
Back_Total OOA	PC6	0.100	0.100
Back_Total OOA	PC7	-0.021	0.021
Back_Total OOA	PC8	0.155	0.155
Back_Total OOA	PC9	0.099	0.099
Back_Total OOA	PC10	0.031	0.031
Back_Total OOA	PC11	-0.061	0.061
Back_Total OOA	PC12	0.040	0.040
Back_Total OOA	PC13	0.052	0.052
Back_Total passes	PC1	-0.134	0.134
Back_Total passes	PC2	-0.447	0.447
Back_Total passes	PC3	-0.037	0.037
Back_Total passes	PC4	0.316	0.316
Back_Total passes	PC5	-0.104	0.104
Back_Total passes	PC6	0.133	0.133
Back_Total passes	PC7	-0.263	0.263
Back_Total passes	PC8	0.026	0.026
Back_Total passes	PC9	-0.252	0.252
Back_Total passes	PC10	-0.218	0.218
Back_Total passes	PC11	-0.090	0.090
Back_Total passes	PC12	-0.009	0.009
Back_Total passes	PC13	0.105	0.105
Back_Total receipts	PC1	-0.076	0.076
Back_Total receipts	PC2	-0.483	0.483
Back_Total receipts	PC3	0.060	0.060
Back_Total receipts	PC4	0.271	0.271
Back_Total receipts	PC5	-0.007	0.007
Back_Total receipts	PC6	-0.137	0.137
Back_Total receipts	PC7	0.296	0.296
Back_Total receipts	PC8	0.009	0.009
Back_Total receipts	PC9	-0.258	0.258
Back_Total receipts	PC10	-0.189	0.189
Back_Total receipts	PC11	-0.089	0.089
Back_Total receipts	PC12	0.047	0.047

Back_Total receipts	PC13	0.101	0.101
Forward_Carries	PC1	0.003	0.003
Forward_Carries	PC2	0.061	0.061
Forward_Carries	PC3	-0.031	0.031
Forward_Carries	PC4	-0.132	0.132
Forward_Carries	PC5	-0.002	0.002
Forward_Carries	PC6	-0.087	0.087
Forward_Carries	PC7	-0.042	0.042
Forward_Carries	PC8	-0.074	0.074
Forward_Carries	PC9	0.101	0.101
Forward_Carries	PC10	-0.015	0.015
Forward_Carries	PC11	-0.067	0.067
Forward_Carries	PC12	0.014	0.014
Forward_Kick meters	PC1	-0.145	0.145
Forward_Kick meters	PC2	-0.240	0.240
Forward_Kick meters	PC3	0.184	0.184
Forward_Kick meters	PC4	-0.029	0.029
Forward_Kick meters	PC5	0.182	0.182
Forward_Kick meters	PC6	0.135	0.135
Forward_Kick meters	PC7	0.023	0.023
Forward_Kick meters	PC8	0.064	0.064
Forward_Kick meters	PC9	0.070	0.070
Forward_Meters carried	PC1	0.088	0.088
Forward_Meters carried	PC2	-0.012	0.012
Forward_Meters carried	PC3	0.029	0.029
Forward_Meters carried	PC4	-0.172	0.172
Forward_Meters carried	PC5	-0.023	0.023
Forward_Meters carried	PC6	0.039	0.039
Forward_Meters carried	PC7	-0.018	0.018
Forward_Meters carried	PC8	-0.062	0.062
Forward_Meters carried	PC9	0.131	0.131
Forward_Meters carried	PC10	0.040	0.040
Forward_Meters carried	PC11	0.028	0.028
Forward_Meters carried	PC12	0.033	0.033
Forward_Total complete tackles	PC1	0.035	0.035
Forward_Total complete tackles	PC2	0.068	0.068
Forward_Total complete tackles	PC3	0.030	0.030
Forward_Total complete tackles	PC4	-0.103	0.103
Forward_Total complete tackles	PC5	0.009	0.009
Forward_Total complete tackles	PC6	-0.072	0.072
Forward_Total complete tackles	PC7	-0.054	0.054
Forward_Total complete tackles	PC8	0.004	0.004
Forward_Total complete tackles	PC9	0.040	0.040
Forward_Total complete tackles	PC10	0.021	0.021
Forward_Total complete tackles	PC11	-0.049	0.049
Forward_Total complete tackles	PC12	0.009	0.009
Forward_Total kicks	PC1	-0.244	0.244
Forward_Total kicks	PC2	0.150	0.150
Forward_Total kicks	PC3	-0.164	0.164
Forward_Total kicks	PC4	0.117	0.117
Forward_Total kicks	PC5	-0.063	0.063
Forward_Total kicks	PC6	0.011	0.011

Forward_Total kicks	PC7	-0.035	0.035
Forward_Total kicks	PC8	-0.027	0.027
Forward_Total kicks	PC9	0.148	0.148
Forward_Total OOA	PC1	-0.110	0.110
Forward_Total OOA	PC2	0.009	0.009
Forward_Total OOA	PC3	0.087	0.087
Forward_Total OOA	PC4	0.062	0.062
Forward_Total OOA	PC5	-0.057	0.057
Forward_Total OOA	PC6	-0.118	0.118
Forward_Total OOA	PC7	0.006	0.006
Forward_Total OOA	PC8	-0.023	0.023
Forward_Total OOA	PC9	0.025	0.025
Forward_Total OOA	PC10	0.057	0.057
Forward_Total OOA	PC11	-0.002	0.002
Forward_Total passes	PC1	0.065	0.065
Forward_Total passes	PC2	-0.027	0.027
Forward_Total passes	PC3	0.065	0.065
Forward_Total passes	PC4	-0.104	0.104
Forward_Total passes	PC5	0.013	0.013
Forward_Total passes	PC6	0.031	0.031
Forward_Total passes	PC7	-0.071	0.071
Forward_Total passes	PC8	-0.102	0.102
Forward_Total passes	PC9	0.058	0.058
Forward_Total passes	PC10	0.040	0.040
Forward_Total passes	PC11	-0.010	0.010
Forward_Total receipts	PC1	0.053	0.053
Forward_Total receipts	PC2	0.071	0.071
Forward_Total receipts	PC3	0.076	0.076
Forward_Total receipts	PC4	-0.107	0.107
Forward_Total receipts	PC5	0.021	0.021
Forward_Total receipts	PC6	-0.088	0.088
Forward_Total receipts	PC7	-0.069	0.069
Forward_Total receipts	PC8	-0.083	0.083
Forward_Total receipts	PC9	0.119	0.119
Forward_Total receipts	PC10	0.004	0.004
Forward_Total receipts	PC11	-0.087	0.087
